# Long-read sequencing and *de novo* genome assembly of *Ammopiptanthus nanus*, a desert shrub

**DOI:** 10.1093/gigascience/giy074

**Published:** 2018-06-28

**Authors:** Fei Gao, Xue Wang, Xuming Li, Mingyue Xu, Huayun Li, Merhaba Abla, Huigai Sun, Shanjun Wei, Jinchao Feng, Yijun Zhou

**Affiliations:** 1 College of Life and Evironmental Sciences, Minzu University of China, 27 Zhongguancun South Street, Beijing, 100081, China; 2 Biomarker Technologies Corporation, Floor 8, Shunjie Building, 12 Fuqian Road, Nanfaxin Town, Shunyi District, Beijing, 101300, China; 3 Annoroad Genomics, Building B1, Yard 88, Kechuang six Road, Beijing Economic-Technological Development Area, Fengtai District, Beijing, 100176, China

**Keywords:** *Ammopiptanthus nanus*, PacBio sequencing, genome assembly, genome annotation

## Abstract

**Background:**

*Ammopiptanthus nanus* is a rare broad-leaved shrub that is found in the desert and arid regions of Central Asia. This plant species exhibits extremely high tolerance to drought and freezing and has been used in abiotic tolerance research in plants. As a relic of the tertiary period, *A. nanus* is of great significance to plant biogeographic research in the ancient Mediterranean region. Here, we report a draft genome assembly using the Pacific Biosciences (PacBio) platform and gene annotation for *A. nanus*.

**Findings:**

A total of 64.72 Gb of raw PacBio sequel reads were generated from four 20-kb libraries. After filtering, 64.53 Gb of clean reads were obtained, giving 72.59× coverage depth. Assembly using Canu gave an assembly length of 823.74 Mb, with a contig N50 of 2.76 Mb. The final size of the assembled *A. nanus* genome was close to the 889 Mb estimated by *k*-mer analysis. The gene annotation completeness was evaluated using Benchmarking Universal Single-Copy Orthologs; 1,327 of the 1,440 conserved genes (92.15%) could be found in the *A. nanus* assembly. Genome annotation revealed that 74.08% of the *A. nanus* genome is composed of repetitive elements and 53.44% is composed of long terminal repeat elements. We predicted  37,188 protein-coding genes, of which 96.53% were functionally annotated.

**Conclusions:**

The genomic sequences of *A. nanus* could be a valuable source for comparative genomic analysis in the legume family and will be useful for understanding the phylogenetic relationships of the Thermopsideae and the evolutionary response of plant species to the Qinghai Tibetan Plateau uplift.

## Data Description

### Background information

**Figure 1:**
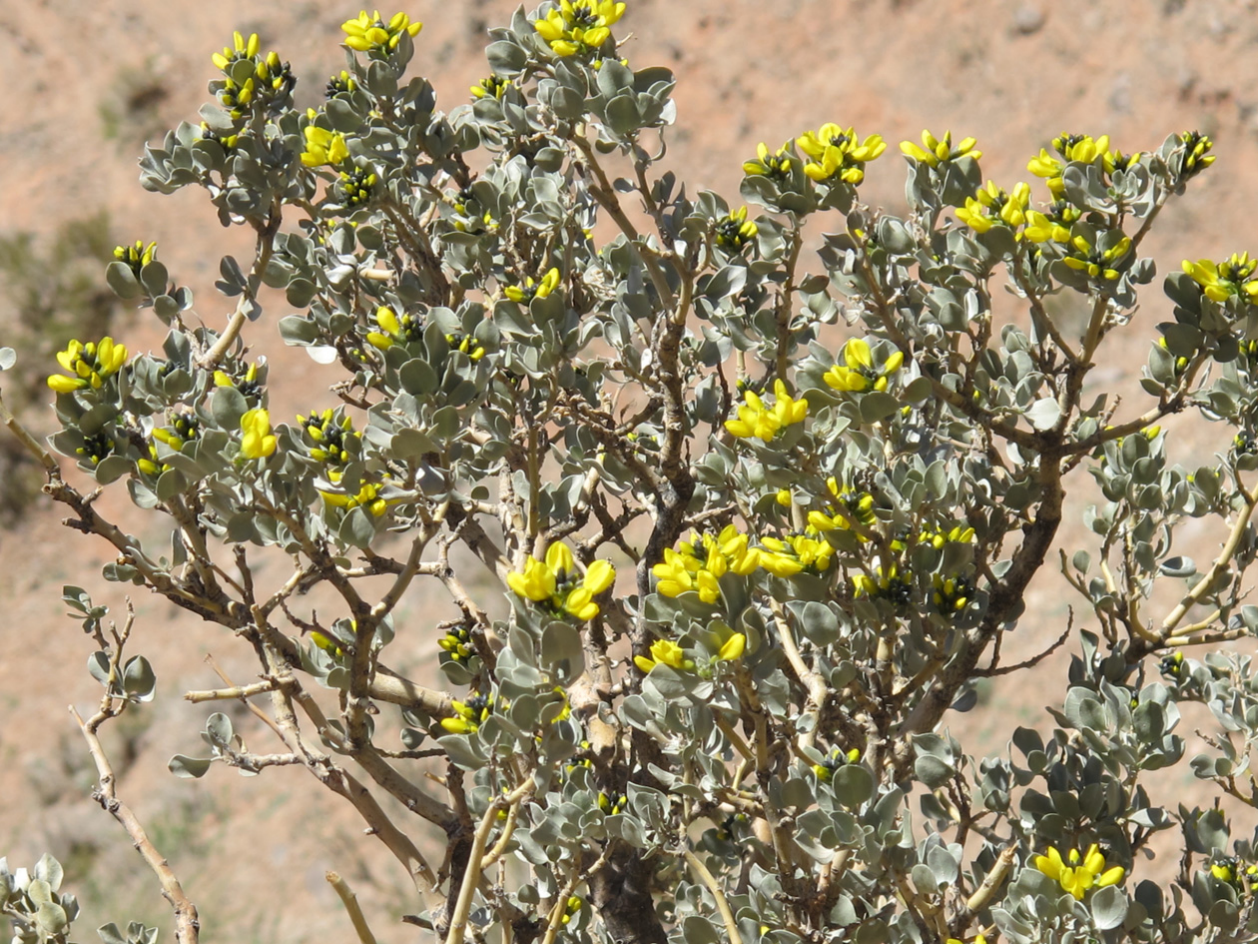
A flowering *A. nanus*.

*Ammopiptanthus nanus*, a desert shrub and a relic from the tertiary period, is one of two species in the genus *Ammopiptanthus*. This genus belongs to the tribe Thermopsideae and the family Fabaceae (Fig. 1). *Ammopiptanthus* is the only genus of evergreen broadleaf shrub distributed in the desert and arid regions of Central Asia. The plants in this genus play important ecological roles by fixing moving sands and delaying further desertification [1].

Tribe Thermopsideae is considered to be a basal branch in the family Fabaceae. The habitats of the ca. 45 plant species in tribe Thermopsideae are interspersed among the Mediterranean Basin, Central Asia, and temperate North America. Studies on the molecular biology of these plant species will promote understanding of the phylogeny of family Fabaceae, as well as some interesting biogeographical topics such as how the Qinghai-Tibetan Plateau uplift and Tethys retreat affected plant evolution [2, 3]. In addition, the genus *Ammopiptanthus* is a unique and isolated branch in tribe Thermopsideae. There are still some debates about the evolution and phylogeny of this genus [3], and more molecular evidence is needed to clarify these issues.

Species in genus *Ammopiptanthus* exhibit extremely high tolerance to drought and freezing and have been used in abiotic tolerance research in plants [4–6]. Although several transcriptome analyses of the response to drought and cold stress have been conducted [1, 7–9], the lack of genome sequence information impedes further investigation into the molecular mechanism underlying the stress tolerance of *Ammopiptanthus* species.

Most of the *de novo* assemblies of plant genomes recently reported have been performed using next-generation sequencing technologies such as Illumina or 454 sequencing platforms [10–12]. However, these assemblies generally contain very fragmented sequences, partly because of the complexity of the plant genome. The newly developed Pacific BioSciences (PacBio) sequencing platform, a third-generation sequencing technology, has started to address some of the intrinsic challenges in sequencing and assembling large and complex plant genomes by producing tens of thousands of long individual reads (up to ∼40 kb) [13]. Recently, several complicated plant genomes, including those of maize [14], sunflower [15], and *Chenopodium quinoa* [16], have been sequenced using the PacBio sequencing technology. In the present study, we used single-molecule real-time (SMRT) sequencing developed by PacBio to generate a draft genome assembly for *A. nanus*.

### Sample collection and genomic DNA sequencing

The leaf tissues of a single *A. nanus* tree (National Center for Biotechnology Information [NCBI] taxonomy ID, 111851) were collected from Xinjiang, China. After collection, tissues were immediately transferred into liquid nitrogen and stored until DNA extraction. DNA was extracted using the Cetyltrimethyl Ammonium Bromide (CTAB) method according to the protocol “Preparing *Arabidopsis* Genomic DNA for Size-Selected ∼20 kb SMRTbell™ Libraries” [17]. The quality of the extracted genomic DNA was checked using 1% agarose gel electrophoresis, and the concentration was quantified using a Qubit fluorimeter (Invitrogen, Carlsbad, CA, USA).

Long-read sequencing was performed at Biomarker Technologies Corporation (Beijing, China) with a PacBio Sequel sequencer (Pacific Biosciences, Menlo Park, CA, USA). The SMRT Bell library was prepared using a DNA Template Prep Kit 1.0 (PacBio p/n 100-259-100), and four 20-kb SMRTbell libraries were constructed. Genomic DNA (10 µg) was mechanically sheared using a Covaris g-Tube (Kbiosciences p/n 520079) with a goal of DNA fragments of approximately 20 kb. A Bioanalyzer 2100 12K DNA Chip assay (Agilent p/n 5067-1508) was used to assess the fragment size distribution. Sheared genomic DNA (5 µg) was DNA-damage repaired and end-repaired using polishing enzymes. A blunt-end ligation reaction followed by exonuclease treatment was conducted to generate the SMRT Bell template. A Blue Pippin device (Sage Science, Inc., Beverly, MA, USA) was used to size select the SMRT Bell template and enrich large fragments (>10 kb). The size-selected library was quality inspected and quantified on an Agilent Bioanalyzer 12 kb DNA Chip (Agilent Technologies, Santa Clara, CA, USA) and a Qubit fluorimeter (Invitrogen, Carlsbad, CA, USA). A ready-to-sequence SMRT Bell-Polymerase Complex was created using a Binding Kit 2.0 (PacBio p/n 100-862-200), according to the manufacturer's instructions. The Sequel instrument was programmed to load and sequence the sample on PacBio SMRT cells v3.0 (PacBio p/n 100-171-800), acquiring one movie of 360 min per SMRT cell. The MagBead loading (PacBio p/n 100-125-900) method was used to improve the enrichment of the larger fragments. A total of 13 SMRT cells were processed yielding 64.72 G subread sequences.

For Illumina sequencing, paired-end (PE) libraries with insert sizes of 350 bp were constructed with the standard protocol provided by Illumina (San Diego, CA, USA) and sequenced on an Illumina HiSeq X Ten platform. A total of 55.97 Gb of PE (2 × 150 bp) clean sequences were generated (Supplementary Table S1). These data were used for genome size estimation, correction of genome assembly, and assembly evaluation.

### Genome size estimation

We characterized genome size and heterozygosity using the distribution of *k*-mers of length 19 from the Illumina HiSeq reads (55.97 Gb clean reads from 350 bp insert size library; NCBI SRA accession number, SRX3286209). This analysis was performed using “kmer_freq_stat” software (developed by Biomarker Technologies). The genome size (G) of *A. nanus* was estimated using the following formula: G = *k*-mer number/average *k*-mer depth, where *k*-mer number = total *k*-mers—abnormal *k*-mers (with too low or too high frequency). The highest peak in the *k*-mer distribution curve was found at the *k*-mer depth of 53, with a *k*-mer number of    47,408,863,457 (Supplementary Fig. S1). The peak at depth of more than 106 was a repetitive peak (*k*-mers duplicated because of repetition). Finally, the *A. nanus* genome size was estimated to be 888.92 Mb, the heterozygosity was approximately 0.02%, and the data used in 19-mer analysis was approximately 53× coverage of the genome.

### Genome assembly

The Sequel raw bam files were converted into subreads in fasta format using the standard PacBio SMRT software package (read data are available at the NCBI SRA accession number, SRX3262947). Then, subreads of less than 500 bp were filtered out. Finally,  7,918,322 reads and    64,538,018,400 bases (∼ 73× depth) were produced. The average subread length was 8.15 kb with a N50 length of 12.79 kb (Supplementary Table S2). The genome assembly was conducted using Canu software (v1.5) [18] (correctedErrorRate = 0.045, corOutCoverage = 70). The draft genome was polished with Arrow (SMRT link v5.0.1, –minCoverage 15) using all SMRT reads and polished using Pilon v1.22 (Pilon, RRID:SCR_014731) [19] using the Illumina reads with the default settings. Finally, we assembled a genome of 823.74 Mb with 1,099 contigs and contig N50 of 2.76 Mb (Supplementary Table S3).

### Repeat annotation and gene prediction

For repeat detection, four software packages, i.e., LTR-FINDER (v1.0.5) [20], MITE-Hunter (v1.0.0) [21], PILER (v1.0) [22], and RepeatScout v1.0.5 (RepeatScout, RRID:SCR_014653) [23], were used to build a *de novo* repeat library on the basis of our assembly with the default settings. Then, the predicted repeats were classified using PASTEClassifier (v1.0) [24] and merged with Repbase (19.06) [25]. Finally, using the resulting repeat database as the final repeat library, RepeatMasker v4.0.5 (RepeatMasker, RRID:SCR_012954) [26] was used to identify repetitive sequences in the *A. nanus* genome with the following parameters: “-nolow -no_is -norna -engine wublast.” Overall, approximately 610.25 Mb of repetitive sequences (74.08% of the assembly) were detected, containing 440.18 Mb (53.44% of the assembly) long terminal read elements (Supplementary Table S4).

*Ab initio*-based, homolog-based, and RNA-sequencing (RNA-seq)-based gene prediction methods were used in combination to identify the protein-coding genes in the *A. nanus* genome assembly. Genscan [27], Augustus v2.4 (Augustus, RRID:SCR_008417) [28], GlimmerHMM v3.0.4 (GlimmerHMM, RRID:SCR_002654) [16], GeneID (v1.4) [29], and SNAP v2006-07-28 (SNAP, RRID:SCR_002127) [30] with the default parameters were used for the *ab initio*- based gene prediction, and all of these software packages were trained using the *Arabidopsis* gene model before gene prediction. For gene prediction using Augustus, in addition to the Arabidopsis's gene model, the Program to Assemble Spliced Alignments (PASA's) gene model was also used as the initial gene model for training. Finally, the best gene model with higher accuracy and specificity was used. The quality of the gene models was evaluated by aligning transcriptome sequences to the whole genome assembly using Tophat (Supplementary Table S5). GeMoMa (v1.3.1) [31] was used in homolog-based gene annotation, and the protein databases of *Cicer arietinum* (GCA_000331145.1), *Phaseolus vulgaris* (GCA_000499845.1), *Glycine max* (GCA_000004515.3), and *Arachis duranensis* (GCA_000817695.2) from GenBank were used as the references. For the RNA-seq-based method of gene prediction, TransDecoder (v2.0, [32]), GeneMarkS-T v5.1 (RRID:SCR_011930) [33], and PASA v2.0.2 (RRID:SCR_014656) [34] were used, and the *A. nanus* transcriptome data were assembled in a previous study (NCBI SRA accession numbers, SRX1409432 and SRX1406652) [35]. Finally, the results from the three methods were integrated using EVM (v1.1.1, RRID:SCR_014659) [36]. Higher weights were assigned to the PASA-predicted transcripts from unigenes and GeMoMa-predicted homologous transcripts than to the *ab initio*- predicted transcripts when conducting the EVM integration. In total, a gene set with  37,144 protein-coding genes was predicted from the *A. nanus* genome assembly (Table 1, Supplementary Table S6, Supplementary Fig. S2). These genes were scattered over 1,099 contigs, averaging 33.80 genes per contig. The genes were annotated by aligning to the Non-redundant protein sequences; Nt: Nucleotide collection (NR, Nt), eukaryotic orthologous groups of proteins (KOG) [37], Kyoto Encyclopedia of Genes and Genomes (KEGG) (KEGG, RRID:SCR_001120) [31], Swissprot (Swissprot, RRID:SCR_002380) [38], and TrEMBL [39] databases using the Basic Local Alignment Search Tool (BLAST) with an e-value cutoff of 1E-5 and also aligned to the Pfam (Pfam, RRID:SCR_004726) database [40] using hmmer V3.0 (-E 0.00001 –domE 0.00001 –cpu 2 –noali –acc) [41]. Gene Ontology (GO) terms were assigned to the genes using the BLAST2GO pipeline [42]. In all, 96.71% of the predicted genes could be classified into families according to their putative functions (Table 2).

**Table 1:** Summary of *A. nanus* genome annotation

Method	Software and gene set	Gene number
*Ab initio* based	Genscan	26,702
	Augustus	43,844
	GlimmerHMM	42,368
	GeneID	45,561
	SNAP	55,094
Homology based	GeMoMa	
	*Arachis duranensis*	27,630
	*Cicer arietinum*	29,229
	*Phaseolus vulgaris*	27,554
	*Glycine max*	31,559
RNA-seq based	PASA	43,810
	TransDecoder	68,687
	GeneMarkS-T	44,944
Integration	EVM	37,173

**Table 2:** Summary of functional annotation for the predicted genes

Annotation database	Annotated gene number	Percentage (%)
GO	20,177	54.28
KEGG	10,130	27.25
KOG	18,237	49.06
Pfam	26,727	71.90
Swissprot	21,401	57.57
TrEMBL	34,946	94.01
NR	34,909	93.91
Nt	34,041	91.57
All Annotated	35,950	96.71

For pseudogene prediction, GenBlastA [43] was used to scan the *A. nanus* genome for sequences homologous to the known protein-coding genes it contained. Then, GeneWise (GeneWise, RRID:SCR_015054) [44] was adopted to search the premature stop codons or frameshift mutations in those sequences and, consequently, to identify pseudogenes. In total, 7,891 pseudogenes were identified from the *A. nanus* genome (Supplementary Table S7).

### Assessment of the genome assembly

First, the 55.97 G Illumina sequencing reads (NCBI SRA accession number, SRX3286209) used for *k*-mer analysis were aligned to the *A. nanus* genome assembly using bowtie [45]. The results showed that all Illumina reads were mapped and 98.45% PE reads were mapped concordantly (Supplementary Table S8). Using these short reads, the estimated quality value (QV) of *A. nanus* genome was calculated according to a previously described method [46, 47], and the erroneous bases in the genome assembly were identified using the variant calling software FreeBayes v0.9.14 (FreeBayes, RID: SCR_010761) with default parameters. The QV of the *A. nanus* genome was estimated to be 38.95, which means that the accuracy of the assembly in base level is fine after base correction.

Second, the *A. nanus* unigenes assembled in a previous study (NCBI SRA accession numbers, SRX1409432 and SRX1406652) [35] were aligned to the *A. nanus* genome using the BLAST-like alignment tool v0.36 (BLAT, RRID:SCR_011919) [48] with default parameters. The alignment indicated that 100% of unigene (≥500 bp in length) assemblies were mapped to the *A. nanus* genome assembly (Table 3).

**Table 3:** Alignment of the unigenes to the *A. nanus* genome assembly

Range of length	Total number	Aligned number	Percentage
≥500	81,429	81,429	100
≥1000	54,385	54,385	100

We also evaluated the completeness of the genome assembly of *A. nanus* using Benchmarking Universal Single-Copy Orthologs (BUSCO) v2.0 (BUSCO, RRID:SCR_015008) [49]. The results showed that 9,215% (1,327 out of 1,440 BUSCOs) of plant sets (embryophyta_odb9, download from [50]) were identified as complete in the *A. nanus* assembly (Supplementary Table S9). Together, the results indicated that our dataset represented a genome assembly with a high level of coverage.

## Conclusions

In summary, the draft genome sequence of *A. nanus* that we obtained demonstrated that third-generation sequencing technology, such as the PacBio platform, could be useful in deciphering complex plant genomes. The availability of the *A. nanus* genome sequence should facilitate *de novo* genome assembly of other species in this genus. The datasets from the present study could not only provide a valuable source for further comparative genomics analysis in the legume family and help to answer some important questions related to the biogeography in the ancient Mediterranean region but also could facilitate our understanding of how plants adapt to the stressful conditions in temperate deserts in Central Asia.

## Availability of supporting data

Raw genomic sequence reads are available in the NCBI Sequence Read Archive under project number PRJNA413722. Supporting data are available from the *GigaScience* GigaDB database [51].

## Supplementary Material

GIGA-D-17-00264_Original_Submission.pdf

GIGA-D-17-00264_Revision_1.pdf

GIGA-D-17-00264_Revision_2.pdf

Reviewer_1_Report_(Original_Submission) -- Nevin Young 11/13/2017 Reviewed

Reviewer_2_Report_(Original_Submission) -- Yinping Jiao 11/19/2017 Reviewed

Reviewer_2_Report_(Revision_1) -- Yinping Jiao 01/18/2018 Reviewed

Reviewer_2_Report_(Revision_2) -- Yinping Jiao 05/17/2018 Reviewed

Reviewer_3_Report_(Original_Submission) -- Felix Bemm 11/27/2017 Reviewed

Reviewer_3_Report_(Revision_1) -- Felix Bemm 8/02/2018 Reviewed

Reviewer_3_Report_(Revision_2) -- Felix Bemm 5/15/2018 Reviewed

Additional Files

## Additional files

**Figure S1**. Distribution of k-mers of length 19 from the Illumina Hiseq reads

**Figure S2**. Venn diagram plot using UpSetR showing the overlap of the gene annotation results of the three gene prediction methods

**Table S1**. Illumina sequencing reads used for genome size estimation and correction of genome assembly

**Table S2**. Size distribution of the PacBio subreads

**Table S3**. Statistics of the *A. nanus* assembly

**Table S4**. Statistics of the identified repeat sequences

**Table S5**. Statistics of mapping the transcriptome data to genome assembly

**Table S6**. Statistics of the genome annotation

**Table S7**. Statistics of the predicted pseudogenes

**Table S8**. The alignment of the Illunima reads to the *A. nanus* genome assembly

**Table S9**. BUSCO assessment of the *A. nanus* genome assembly

## Abbreviations

BLAST: Basic Local Alignment Search Tool; BUSCO: Benchmarking Universal Single-Copy Orthologs; GO: Gene Ontology; KEGG: Kyoto Encyclopedia of Genes and Genomes; KOG: eukaryotic orthologous groups of proteins; PacBio: Pacific Biosciences; PASA: PE: paired-end; QV: quality value; RNA-seq: RNA sequencing; SMRT: single-molecule real-time.
