# Supplementary material for: Long-read sequencing and de novo genome assembly of Ammopiptanthus nanus, a desert shrub
Source: Gigascience. 2018 Jun 28;7(7):giy074. doi: 10.1093/gigascience/giy074 (PMC6048559; doi:10.1093/gigascience/giy074)
Supplement: GIGA-D-17-00264_Original_Submission.pdf [file giy074_giga-d-17-00264_original_submission.pdf]

GigaScience

Long-read sequencing and de novo genome assembly of Ammopiptanthus nanus, a desert shrub

--Manuscript Draft--

Manuscript Number:	GIGA-D-17-00264
Full Title:	Long-read sequencing and de novo genome assembly of Ammopiptanthus nanus, a desert shrub
Article Type:	Data Note

Funding Information:

Funder	Recipient
the National Natural Science Foundation of China (31370356)	Prof. Yijun Zhou
the National Natural Science Foundation of China (31670335)	Dr. Fei Gao
the Ministry of Education of China through 111 projects (B08044)	Prof. Yijun Zhou
the Ministry of Education of China through 985 projects (YLDX01013)	Prof. Yijun Zhou

Abstract:

Background

Ammopiptanthus nanus is a rare broad-leaved shrub in the desert and arid regions of Central Asia. This plant species exhibits extremely high tolerance to drought and freezing stresses and has been used in abiotic tolerance research in plant. As a relic of Tertiary period, A. nanus is of great significance to plant biogeographic research in ancient Mediterranean region. Here we report a draft genome assembly using PacBio platform and gene annotation for A. nanus.

Findings

A total of 64.72 gigabases (Gb) of raw PacBio Sequel reads were generated from four 20 kb libraries. After filtering, 64.53 Gb of clean reads were obtained, giving 72.59 × coverage depth. Assembly using Canu gave an assembly length of 823 Mb, with a contig N50 of 2.76 Mb. The final size of the assembled A. nanus genome (823 Mb) was close to the 889 Mb estimated by k-mer analysis. The genome completeness was evaluated by BUSCO, and 1,328 out of the 1,440 conserved genes (92.22%) could be found in the A. nanus assembly. Genome annotation revealed that 74.09% of the A. nanus genome is composed of repetitive elements, 70.71 % of transposable elements, and 53.38 % of long terminal repeat elements (LTRs). We predicted 37,259 protein-coding genes, of which 96.7% were functionally annotated.

Conclusions

The genomic sequences of A. nanus could provide valuable source for comparative genomics analysis in family legume, and will be useful for understanding the phylogenetic relationships of the Thermopsideae and the evolutionary response of plant species to the Qinghai Tibetan Plateau uplift.

Corresponding Author:	Fei Gao<br>Minzu University of China<br>Beijing, CHINA
Corresponding Author Secondary Information:	
Corresponding Author's Institution:	Minzu University of China
Corresponding Author's Secondary Institution:	
First Author:	Fei Gao
First Author Secondary Information:	
Order of Authors:	Fei Gao
	Xue Wang

	Huayun Li
	Jinchao Feng
	Yijun Zhou
	Mingyue Xu
	Shanjun Wei
	Abla Merhaba
	Huigai Sun
**Order of Authors Secondary Information:**	
**Opposed Reviewers:**	
**Additional Information:**	
**Question**	**Response**
Are you submitting this manuscript to a special series or article collection?	No
**Experimental design and statistics**<br><br>Full details of the experimental design and statistical methods used should be given in the Methods section, as detailed in our Minimum Standards Reporting Checklist. Information essential to interpreting the data presented should be made available in the figure legends.<br><br>Have you included all the information requested in your manuscript?	Yes
**Resources**<br><br>A description of all resources used, including antibodies, cell lines, animals and software tools, with enough information to allow them to be uniquely identified, should be included in the Methods section. Authors are strongly encouraged to cite Research Resource Identifiers (RRIDs) for antibodies, model organisms and tools, where possible.<br><br>Have you included the information requested as detailed in our Minimum Standards Reporting Checklist?	Yes
**Availability of data and materials**<br><br>All datasets and code on which the conclusions of the paper rely must be either included in your submission or deposited in publicly available repositories	Yes

Powered by Editorial Manager® and ProduXion Manager® from Aries Systems Corporation

(where available and ethically appropriate), referencing such data using a unique identifier in the references and in the "Availability of Data and Materials" section of your manuscript.<br><br>Have you have met the above requirement as detailed in our Minimum Standards Reporting Checklist?	

Manuscript

Click here to download Manuscript De novo genome assembly of Ammopiptanthus nanus_12.docx

Long-read sequencing and *de novo* genome assembly of *Ammopiptanthus nanus*, a desert shrub

Fei Gao[1], Xue Wang[1], Mingyue Xu[2], Huayun Li[3], Abla Merhaba[1], Huigai Sun[1], Shanjun Wei[1], Jinchao Feng[1*], Yijun Zhou[1*]

[1]College of Life and Environmental Sciences, Minzu University of China, Beijing, 100081, China

[2]Biomarker Technologies Corporation, Beijing, 101300, China.

[3]Annoroad Genomics, Beijing, 100176, China

Email addresses: Fei Gao <gaofei@muc.edu.ecn>, Xue Wang <wangxue@muc.edu.cn>, Mingyue Xu<xumy@biomarker.com.cn>, Huayun Li < huayunli@annoroad.com >, Abla Merhaba< Merhaba@muc.edu.cn >, Huigai Sun <sunhuigai66@163.com>, Shanjun Wei<wei.s.j@163.com>

*Correspondence should be addressed to: Y. Z. <zhouyijun@muc.edu.cn>, J. F. <fengjinchao@muc.edu.cn>

Abstract

Background

Ammopiptanthus nanus is a rare broad-leaved shrub in the desert and arid regions of Central Asia. This plant species exhibits extremely high tolerance to drought and freezing stresses and has been used in abiotic tolerance research in plant. As a relic of Tertiary period, *A. nanus* is of great significance to plant biogeographic research in ancient Mediterranean region. Here we report a draft genome assembly using PacBio platform and gene annotation for *A. nanus*.

Findings

A total of 64.72 gigabases (Gb) of raw PacBio Sequel reads were generated from four 20 kb libraries. After filtering, 64.53 Gb of clean reads were obtained, giving 72.59 × coverage depth. Assembly using Canu gave an assembly length of 823 Mb, with a contig N50 of 2.76 Mb. The final size of the assembled *A. nanus* genome (823 Mb) was close to the 889 Mb estimated by k-mer analysis. The genome completeness was evaluated by BUSCO, and 1,328 out of the 1,440 conserved genes (92.22%) could be found in the *A. nanus* assembly. Genome annotation revealed that 74.09% of the *A. nanus* genome is composed of

repetitive elements, 70.71 % of transposable elements, and 53.38 % of long terminal repeat elements (LTRs). We predicted 37,259 protein-coding genes, of which 96.7% were functionally annotated.

Conclusions

The genomic sequences of *A. nanus* could provide valuable source for comparative genomics analysis in family legume, and will be useful for understanding the phylogenetic relationships of the Thermopsideae and the evolutionary response of plant species to the Qinghai Tibetan Plateau uplift.

Keywords

Ammopiptanthus nanus, PacBio sequencing, Genome assembly, Genome annotation

Data Description

Background information

Ammopiptanthus nanus, a desert shrub and a relic from the tertiary period, is one of two species in genus *Ammopiptanthus* and this genus belongs to tribe Thermopsideae, family Fabaceae (Figure 1). *Ammopiptanthus* is the only genus of evergreen broadleaf shrub distribute in the desert and arid regions of Central Asia, and the plants in this genus play important ecological roles by fixing moving sands and delaying further desertification [1].

Tribe Thermopsideae is considered to be a basal branch in the family Fabaceae and the habitats of the ca. 45 plant species in Tribe Thermopsideae intersperse among the Mediterranean Basin, central Asia and temperate North America. Studies on the molecular biology of these plant species will promote understanding of phylogeny of family Fabaceae, and of some interesting biogeographical topics, such as how Qinghai-Tibetan Plateau uplift and Tethys retreat affect plant evolution [2, 3]. In addition, genus *Ammopiptanthus* is a unique and isolate branch in tribe Thermopsidea. There are still some debate about the evolution and phylogeny of this genus [3], and more molecular evidence is needed to clarify those issues.

The plant species in Genus *Ammopiptanthus* exhibit extremely high tolerance to drought and freezing stresses and has been used in abiotic tolerance research in plant. Although several transcriptome analysis of

the response to drought and cold stress have been conducted [1, 4–6], the lack of genome sequence information impedes the further investigation into the molecular mechanism underlying the stress tolerance of *Ammopiptanthus* species.

Most of the *de novo* assemblies of plant genomes reported recently have been performed using the next generation sequencing technologies such as Illumina or 454 sequencing platforms [7–9]. However, those assemblies generally have a low N50 values and a large number of contigs, partly because of the complexity of the plant genome. The newly developed Pacific BioSciences (PacBio) sequencing platform, a third-generation sequencing technology, has started to address some of the intrinsic challenges in sequencing and assembling large and complex plant genomes, via producing tens of thousands of long individual reads (up to ~40 kb) [10]. In the present study, we employed single molecule real-time (SMRT) sequencing developed by PacBio, to generate a draft genome assembly for *A. nanus*.

Sample collection and genomic DNA sequencing

The leaf tissues of a single *A. nanus* tree (NCBI Taxonomy ID: 111851) were collected from Xinjiang, China. After collection, tissues were immediately transferred into liquid nitrogen and stored until DNA extraction. The extraction of DNA was conducted using CTAB method according to the protocol ‘Preparing Arabidopsis Genomic DNA for Size-Selected ~20 kb SMRTbell™ Libraries’ (http://www.pacb.com/wp-content/uploads/2015/09/Shared-Protocol-Preparing-Arabidopsis-DNA-for-20-kb-SMRTbell-Libraries.pdf). The quality of the extracted genomic DNA was checked by 1% agarose gel electrophoresis, and the concentration was quantified using a Qubit fluorimeter (Invitrogen, Carlsbad, CA, USA).

Long-read sequencing was performed at Biomarker Technologies Corporation (Beijing, China) with a PacBio Sequel sequencer (Pacific Biosciences, Menlo Park, CA, USA). The SMRT Bell library was prepared using a DNA Template Prep Kit 1.0 (PacBio p/n 100-259-100) and four 20 kb SMRTbell libraries were constructed. Genomic DNA (10 µg) was mechanically sheared using a Covaris gTube (Kbiosciences p/n 520079) aiming at DNA fragments of about 20 kb. A Bioanalyzer 2100 12K DNA Chip assay (Agilent

p/n 5067-1508) was used to assess the fragment size distribution. Sheared genomic DNA (5 μg) was DNA-damage repaired and end-repaired using polishing enzymes. A blunt-end ligation reaction followed by exonuclease treatment was conducted to generate the SMRT Bell template. A Blue Pippin device (Sage Science, Inc., Beverly, MA, USA) was used to size select the SMRT Bell template and enrich large fragments (> 10 kb). The size-selected library was quality inspected and quantified on Agilent Bioanalyzer 12 kb DNA Chip and Qubit Fluorimeter (Life Technologies). A ready-to-sequence SMRT Bell-Polymerase Complex was created using a Binding Kit 2.0 (PacBio p/n 100-862-200), according to the manufacturer's instructions. The Sequel instrument was programmed to load and sequence the sample on PacBio SMRT cells v3.0 (PacBio p/n 100-171-800), acquiring one movie of 360 min per SMRT cell. The MagBead loading (PacBio p/n 100-125-900) method was employed in order to improve the enrichment of the larger fragments. A total of 13 SMRT cells were processed yielding 64.72 G subread sequences.

For Illunima sequencing, paired-end libraries with insert sizes of 350 bp were constructed with the standard protocol provided by Illumina (San Diego, CA, USA) and sequenced on an Illumina HiSeq X ten platform. A total of 55.97 Gb of paired-end (2×150 bp) clean sequences were generated (Table S1). These data were used for genome size estimation, correction of genome assembly, and assembly evaluation.

Genome size estimation

We characterized the genome size and heterozygosity using the distribution of k-mers of length 19 from the Illumine Hiseq reads (55.97 Gb clean reads from 350 bp insert size library). This analysis was performed using "kmer_freq_stat" software (developed by Biomarker Technologies). The genome size (G) of *A. nanus* was estimated by the following formula: G = k-mer number/average k-mer depth, here, k-mer number = Total k-mers - abnormal k-mers (with too low or too high frequency). The highest peak in at the k-mer distribution curve was found at k-mer depth of 53, with a k-mer number of 47,408,863,457 (Figure S1). The peak at depth of more than 106 was a repetitive peak (k-mers duplicated due to repetition). Finally, the *A. nanus* genome size was estimated to be 888.92 Mb, the heterozygosity is approximately 0.02%, and the data used in 19-mer analysis was about 53× coverage of the genome.

Genome assembly

The Sequel raw bam files were converted into subreads in fasta format using the standard PacBio SMRT software package. Then subreads of shorter than 500 bp were filtered out. Finally, 7,918,322 reads and 64,538,018,400 bases (~ 73 × depth) were produced. The average subread length was 8.15 kb with a N50 length of 12.79 kb (Table 1 and S2). The genome assembly was conducted using Canu software (v1.5) [11] (correctedErrorRate=0.045, corOutCoverage=70). Then, the resulting draft genome was polished by Pilon (v1.22, RRID:SCR_014731) [12] using the default settings to correct sequencing errors in genome assembly. Finally, we assembled a genome of 823 Mb with 1,099 contigs and contig N50 of 2.76 Mb (Table 2).

Repeat annotation and gene prediction

For repeat detection, first, four software, i.e., LTR-FINDER (v1.0.5) [13], MITE-Hunter (v1.0.0) [14], PILER (v1.0) [15], and RepeatScout (v1.0.5, RRID:SCR_014653) [16] were used to build a *de novo* repeat library on the basis of our assembly with the default settings, and then, the predicted repeats were classified using PASTEClassifier (v1.0) [17] and merged with Repbase (19.06) [18], finally, using the resulting repeat database as the final repeat library, RepeatMasker (v4.0.5; RepeatMasker, RRID:SCR 012954) [19] was utilized to identify repetitive sequences in *A. nanus* genome with the following parameters "-nolow -no_is -norna -engine wublast". Overall, approximately 610.22 Mb of repetitive sequences (74.09% of the assembly) were detected, containing 439.66 Mb (53.38% of the assembly) LTRs (Table S3).

Ab initio based, homolog based and RNA-seq based gene prediction methods were conducted in combination to identify the protein-coding genes in the *A. nanus* genome assembly. Genscan [20], Augustus (v2.4, RRID:SCR_008417) [21], GlimmerHMM (v3.0.4, RRID:SCR_002654) [16], GeneID (v1.4) [22], and SNAP (v2006-07-28, RRID:SCR_002127) [23] with the default parameters were employed for the *Ab initio* based gene prediction. GeMoMa (v1.3.1) [24] was used in homolog based gene annotation and protein database of *Cicer arietinum* (GCA_000331145.1), *Phaseolus vulgaris* (GCA_000499845.1), *Glycine max* (GCA_000004515.3), and *Arachis duranensis* (GCA_000817695.2) from GenBank were used as the reference databases. For RNA-seq based method gene prediction, TransDecoder (v2.0,

http://transdecoder.github.io), GeneMarkS-T (v5.1, RRID:SCR_011930) [25], and PASA (v2.0.2, RRID:SCR_014656) [26] were used. Finally, the results from the three methods were integrated using EVM (v1.1.1, RRID:SCR_014659) [27]. Totally, a gene set with 37,259 protein-coding genes was predicted from the *A. nanus* genome assembly (Table 3 and 4, and Figure 2). These genes were scattered over 1,099 contigs, averaging 33.90 genes per contig. By alignment to NR, Nt, KOG [28], GO [29], KEGG (RRID:SCR_001120) [30], Swissprot (RRID:SCR_002380) [31], TrEMBL [31], and Pfam database (RRID:SCR_004726) [32] using blast with an e-value cutoff of 1E-5, 96.70% of the predicted genes could be functionally annotated (Table 5).

For pseudogene prediction, first, GenBlastA [33] was used to scan the *A. nanus* genome for sequences homologous to the known protein-coding genes it contains, then GeneWise (RRID:SCR_015054) [34] was adopted to search the premature stop codon or frameshift mutation in those sequences and, consequently, to identify pseudogenes. In total, 7,588 pseudogenes were identified from the *A. nanus* genome (Table 6).

Completeness of the genome assembly

First, the 55.97G Illunima sequencing reads used for k-mer analysis were aligned to the *A. nanus* genome assembly using bowtie [35]. The result showed that all Illunima reads were mapped and 98.07% PE reads were mapped concordantly (Table 7).

Second, the *A. nanus* unigenes assembled in a previous study were aligned to the *A. nanus* genome using BLAT v0.36 (BLAT, RRID:SCR_011919) [36] with default parameters. The alignment indicated that 100% of unigenes (> 500 bp in length) assemblies mapped to the *A. nanus* genome assembly (Table 8).

We also evaluated the completeness of the genome assembly of *A. nanus* by using BUSCO v2.0 (BUSCO, RRID:SCR_015008) [37]. The results showed that 92.22% (1,328 out of 1,440 BUSCOs) of plants set (embryophyta_odb9, download from http://busco.ezlab.org/) were identified as complete in the *A. nanus* assembly (Table 9). Together, the results indicated that our dataset represented a genome assembly with a high level of coverage.

Conclusion

In summary, the draft genome sequence of *A. nanus* obtained in the present study shows that third-generation sequencing technology like PacBio platform could be useful in deciphering complex plant genomes. The availability of the *A. nanus* genome sequence should facilitate *de novo* genome assembly of other species in this genus. The datasets from the present manuscript could not only provide valuable source for further comparative genomics analysis in family legume, help to answer some important questions related to the biogeography research in ancient Mediterranean region, but also facilitate understanding of how plant adapt to the stressful conditions in temperate deserts in Central Asia.

List of abbreviations

Gb: Giga base; TE: Transposable element; GO: Gene Ontology; PE: paired-end; LTR: Long terminal repeat element; CDS: Coding DNA sequence; SMRT: Single molecule real-time; PacBio: Pacific BioSciences; KOG: Eukaryotic Orthologous Groups of proteins; KEGG: Kyoto Encyclopedia of Genes and Genomes

Competing interests

The authors declare that they have no competing interests.

Funding

This work was financially supported by the National Natural Science Foundation of China (31370356 and 31670335) and the Ministry of Education of China through 111 and 985 projects (B08044, YLDX01013).

Availability of Supporting Information

Raw genomic sequence reads are available in the NCBI Sequence Read Archive under project number PRJNA413722. Supporting data are also available from the GigaScience database (GigaDB) [38].

Author Contributions

Y.Z and J.F oversaw the project. A.M. X.W. and S.W. collected the sample and extracted the genomic DNA. H.L., M.X., and H.S performed the genome assembly, annotated the genome and analyzed data. F.G analyzed data. F.G and Y.Z wrote the manuscript.

References

1. Gao F, Wang J, Wei S, Li Z, Wang N, Li H. Transcriptomic analysis of drought stress responses in *Ammopiptanthus mongolicus* leaves using the RNA-Seq technique. Plos ONE. 2015;10(4):e0124382.
2. Zhang ML, Huang JF, Sanderson SC, Yan P, Wu YH, Pan BR. Molecular biogeography of tribe Thermopsideae (Leguminosae): A Madrean-Tethyan disjunction pattern with an African origin of core Genistoides. Biomed Res Int. 2015; 2015:864804.
3. Shi W, Liu PL, Duan L, Pan BR, Su ZH. Evolutionary response to the Qinghai-Tibetan Plateau uplift: phylogeny and biogeography of *Ammopiptanthus* and tribe Thermopsideae (Fabaceae). Peer J. 2017;5:e3607.
4. Zhou Y, Gao F, Liu R, Feng J, Li H. *De novo* sequencing and analysis of root transcriptome using 454 pyrosequencing to discover putative genes associated with drought tolerance in *Ammopiptanthus mongolicus*. BMC Genomics. 2012;13:266.
5. Wu Y, Wei W, Pang X, Wang X, Zhang H, Dong B, et al. Comparative transcriptome profiling of a desert evergreen shrub, *Ammopiptanthus mongolicus*, in response to drought and cold stresses. BMC Genomics. 2014;15:671.
6. Pang T, Ye CY, Xia X, Yin W. *De novo* sequencing and transcriptome analysis of the desert shrub, *Ammopiptanthus mongolicus*, during cold acclimation using Illumina/Solexa. BMC Genomics. 2013;14:488.
7. Fu Y, Li L, Hao S, Guan R, Fan G, Shi C, et al. Draft genome sequence of the Tibetan medicinal herb Rhodiola crenulata. Gigascience. 2017;6(6):1–5.
8. Zhao D, Hamilton JP, Pham GM, Crisovan E, Wiegert-Rininger K, Vaillancourt B, et al. *De novo* genome assembly of *Camptotheca acuminata*, a natural source of the anti-cancer compound camptothecin. Gigascience. 2017;6(9):1–7.

9. Xia EH, Zhang HB, Sheng J, Li K, Zhang QJ, Kim C, et al. The tea tree genome provides insights into tea flavor and independent evolution of caffeine biosynthesis. Mol Plant. 2017;10(6):866–77.

10. Roberts RJ, Carneiro MO, Schatz MC. The advantages of SMRT sequencing. Genome Biol. 2013;14(7):405.

11. Koren S, Walenz BP, Berlin K, Miller JR, Bergman NH, Phillippy AM. Canu: scalable and accurate long-read assembly via adaptive k-mer weighting and repeat separation. Genome Res. 2017;27(5):722–36.

12. Walker BJ, Abeel T, Shea T, Priest M, Abouelliel A, Sakthikumar S, et al. Pilon: an integrated tool for comprehensive microbial variant detection and genome assembly improvement. PLoS ONE. 2014;9(11):e112963.

13. Xu Z, Wang H. LTR˙FINDER: an efficient tool for the prediction of full-length LTR retrotransposons. Nucleic Acids Res 2007;35(web server issue):W265–8.

14. Han Y, Wessler SR. MITE-Hunter: a program for discovering miniature inverted-repeat transposable elements from genomic sequences. Nucleic Acids Res. 2010;38(22):e199.

15. Edgar RC, Myers EW. PILER: identification and classification of genomic repeats. Bioinformatics. 2005;21:i152–8.

16. Price AL, Jones NC, Pevzner PA. *De novo* identification of repeat families in large genomes. Bioinformatics 2005;21 (suppl 1):i351–8.

17. Hoede C, Arnoux S, Moisset M, Chaumier T, Inizan O, Jamilloux V, et al. PASTEC: An automatic transposable element classification tool. PLoS ONE. 2014;9:e91929.

18. Bao W, Kojima KK, Kohany O. Repbase Update, a database of repetitive elements in eukaryotic genomes. Mobile DNA. 2015;6:11.

19. Tarailo-Graovac M, Chen N. Using RepeatMasker to identify repetitive elements in genomic sequences. Curr Protoc Bioinformatics. 2009; Chapter 4: Unit 4.10.

20. Burge C, Karlin S. Prediction of complete gene structures in human genomic DNA. J Mol Biol. 1997; 268:78–94.

21. Stanke M, Waack S. Gene prediction with a hidden Markov model and a new intron submodel. Bioinformatics. 2003;19 Suppl 2:ii215–25.

22. Blanco E, Parra G, Guigó R: Using geneid to identify genes. Curr Protoc Bioinformatics. 2007;4.3.

23. Korf I. Gene finding in novel genomes. BMC bioinformatics. 2004;5:59.

24. Keilwagen J, Wenk M, Erickson JL, Schattat, MH, Jan, G, Frank, H. Using intron position conservation for homology-based gene prediction. Nucleic Acids Res. 2016;44:e89.

25. Tang S, Lomsadze A, Borodovsky M. Identification of protein coding regions in RNA transcripts. Nucleic Acids Res. 2015;43(12):e78.

26. Campbell MA, Haas BJ, Hamilton JP, Mount SM, Buell CR. Comprehensive analysis of alternative splicing in rice and comparative analyses with *Arabidopsis*. BMC genomics. 2006;7:327.

27. Haas BJ, Salzberg SL, Zhu W, Pertea M, Allen JE, Orvis J, et al. Automated eukaryotic gene structure annotation using EVidenceModeler and the Program to Assemble Spliced Alignments. Genome Biol. 2008;9(1):R7.

28. Tatusov RL, Natale DA, Garkavtsev IV, Tatusova TA, Shankavaram UT, Rao BS, et al. The COG database: new developments in phylogenetic classification of proteins from complete genomes. Nucleic Acids Res. 2001;29(1):22–8.

29. Dimmer EC, Huntley RP, Alam-Faruque Y, Sawford T, O'Donovan C, Martin MJ, et al. The UniProt-GO annotation database in 2011. Nucleic Acids Res. 2012:40(Database issue):D565–70.

30. Kanehisa M, Goto S. KEGG: Kyoto Encyclopedia of Genes and Genomes. Nucleic Acids Res 2000;28(1):27–30.

31. Boeckmann B, Bairoch A, Apweiler R, Blatter M-C, Estreicher A, Gasteiger E, et al. The SWISS-PROT protein knowledgebase and its supplement TrEMBL in 2003. Nucleic Acids Res. 2003;31(1):365–70.

32. Zdobnov EM, Apweiler R. InterProScan–an integration platform for the signature-recognition methods in InterPro. Bioinformatics. 2001;17:847–8.

33. She R, Chu JS, Wang K, Pei J, Chen N. GenBlastA: enabling BLAST to identify homologous gene sequences. Genome Res. 2009;19(1):143–9.

34. Birney E, Clamp M, Durbin R. GeneWise and genomewise. Genome Res. 2004;14(5):988–95.

35. Langmead B, Trapnell C, Pop M, Salzberg SL. Ultrafast and memory-efficient alignment of short DNA sequences to the human genome. Genome Biol. 2009;10(3):R25

36. Kent WJ. BLAT–the BLAST-like alignment tool. Genome Res. 2002;12(4):656–64.

37. Simao FA, Waterhouse RM, Ioannidis P, Kriventseva EV, Zdobnov EM. BUSCO: assessing genome assembly and annotation completeness with single-copy orthologs. Bioinformatics. 2015;31(19):3210–2.

38. Supporting data for "Long-read sequencing and *de novo* genome assembly of *Ammopiptanthus nanus*, a desert shrub ". GigaScience Database. 2017. http://dx.doi.org/**.****/******

Tables

Table 1. PacBio subreads used for the *A. nanus* genome assembly.

Type	Read bases (bp)	Reads number	Read N50 (bp)	Mean length (bp)
Subread	64,538,018,400	7,918,322	12,786	8,150

Table 2. Statistics of the *A. nanus* assembly

Contig number	Contig length (bp)	Contig N50 (bp)	Contig N90 (bp)	Contig max (bp)	GC content (%)	Gap total length (bp)
1,099	823,653,484	2,761,160	562,763	11,314,623	36.74	0

Table 3 Summary of *A. nanus* genome annotation

Method	Software and gene set	Gene number
Ab initio based	Genscan	26,686
	Augustus	32,931
	GlimmerHMM	43,252
	GeneID	45,622
	SNAP	58,443
Homology based	GeMoMa	
	Arachis duranensis	98,866
	Cicer arietinum	54,840
	Phaseolus vulgaris	56,919
	Glycine max	97,568
RNA-seq based	PASA	43,789
	TransDecoder	43,215
	GeneMarkS-T	30,748
Integration	EVM	37,259

Table 4 Statistics of the genome annotation

Gene number	Gene Length (bp)	Avery gene length (bp)	CDS length (bp)	Avery CDS length (bp)	Intron length (bp)	Avery intron length (bp)
37,259	173,484,514	4,656.18	42,040,206	1,128.32	118,059,238	3168.61

Table 5 Summary of functional annotation for the predicted genes

Annotation database	Annotated gene number	Percentage (%)
GO	20,236	54.31
KEGG	10,169	27.29
KOG	18,294	49.09
Pfam	26,793	71.91
Swissprot	21,461	57.59
TrEMBL	35,021	93.99
NR	34,984	93.89
Nt	34,124	91.58
All Annotated	36,033	96.70

Table 6 Statistics of the predicted pseudogenes

Pseudogene number	Total length (bp)	Average length (bp)
7,588	20,322,021	2,678.17

Table 7 The alignment of the Illunima reads to the *A. nanus* genome assembly

Library	Total reads	Mapped (%)	Concordantly mapped (%)
350 bp	373,513,096	100	98.07

Table 8 The alignment of the unigenes to the *A. nanus* genome assembly

Range of length	Total number	Aligned number	Percentage (%)
>=500	81,429	81,429	100
>=1,000	54,385	54,385	100

Table 9 BUSCO assessment of the *A. nanus* genome assembly

Complete BUSCOs	Complete and single-copy BUSCOs	Complete and duplicated BUSCOs	Fragmented BUSCOs	Missing BUSCOs
1,328	1,239	89	35	77

Figure legends

Figure 1. A flowering *A. nanus*

Figure 2. Venn diagram showing the overlap of the gene annotation results of the three categories of gene prediction methods

Figure 1

Click here to download Figure Figure_1.jpg

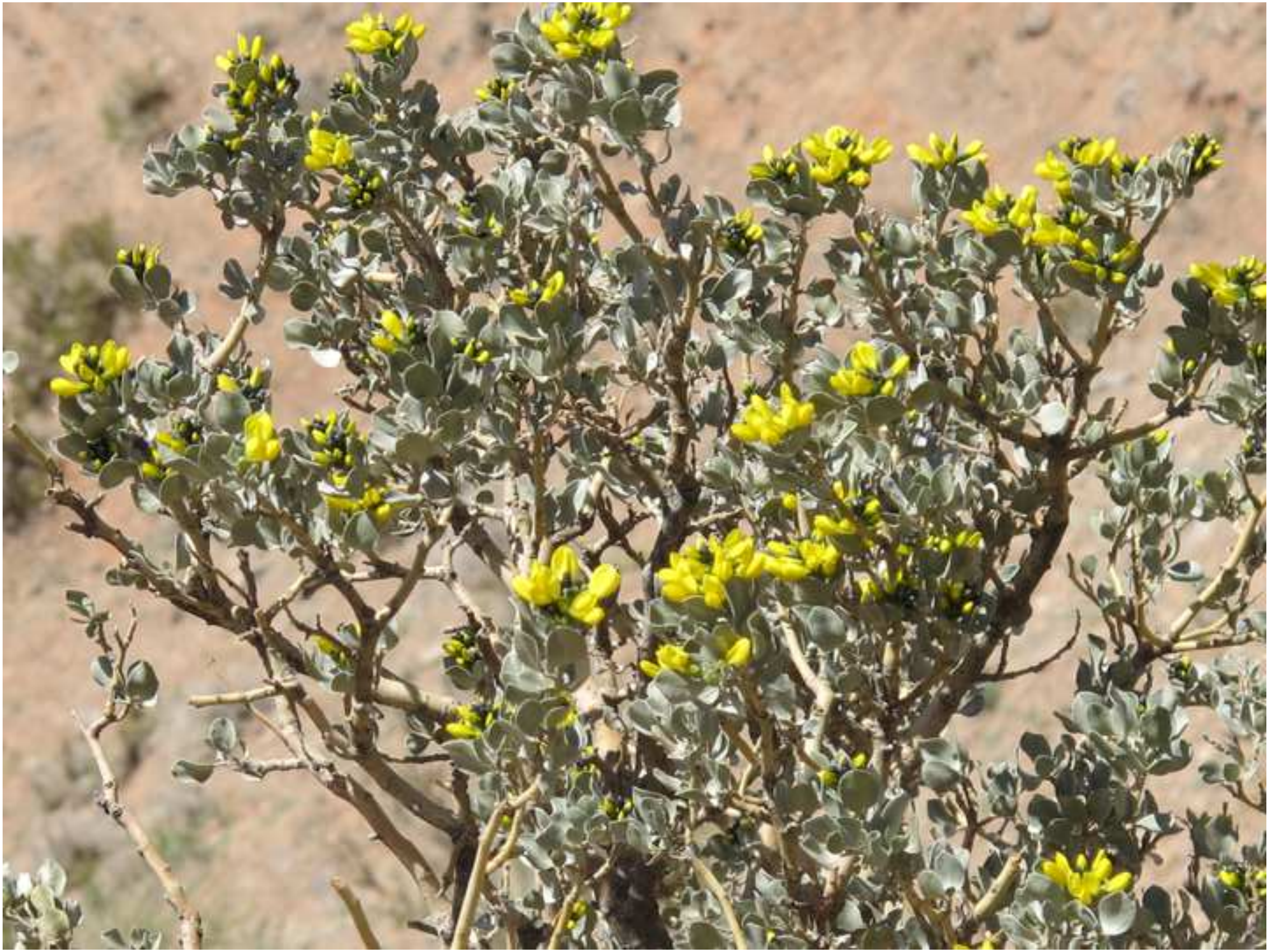

Figure 2

Click here to download Figure Fig_2.tif

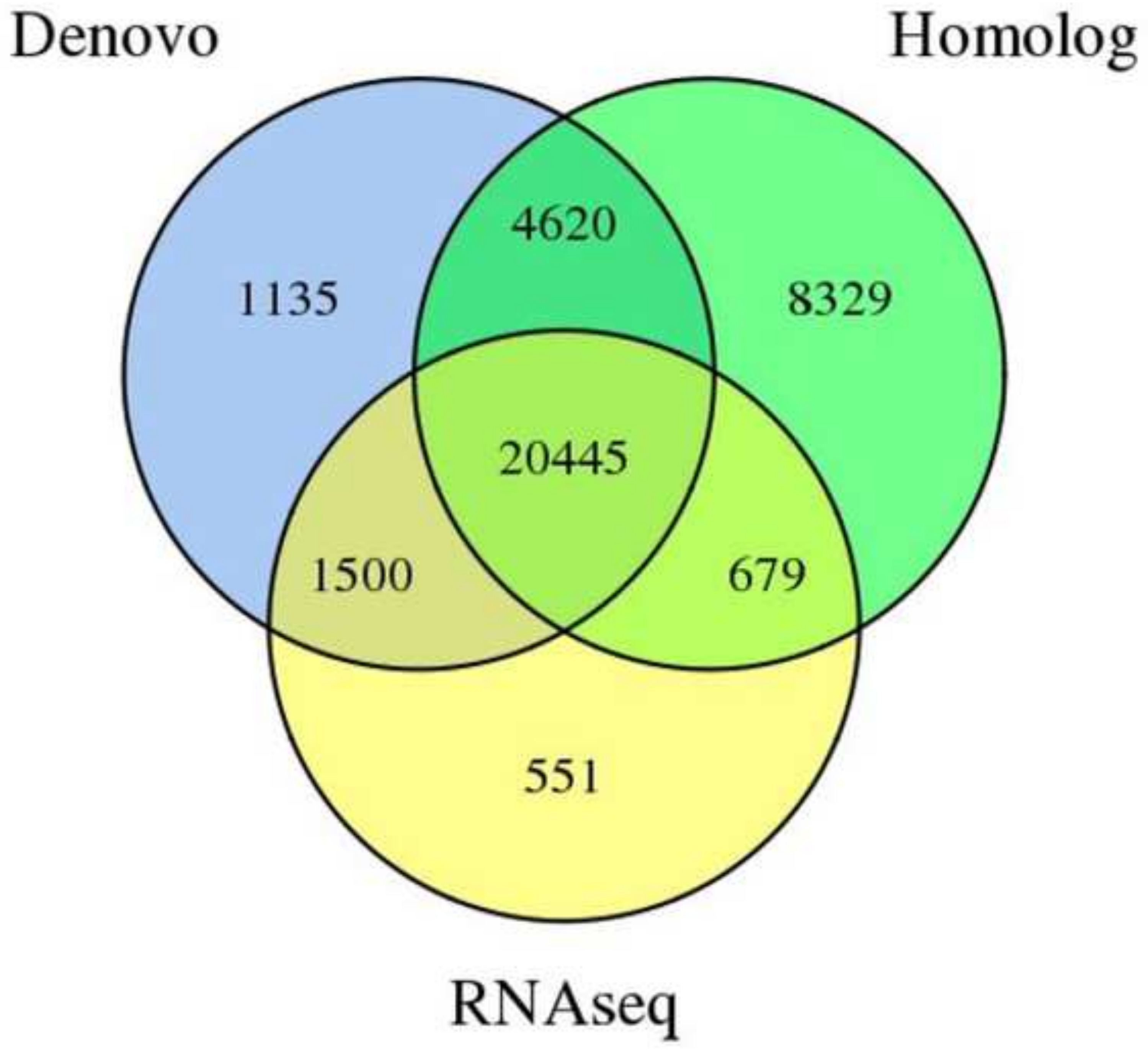

Click here to access/download
Supplementary Material
Supplementary material.docx
