# Supplementary material for: Long-read sequencing and de novo genome assembly of Ammopiptanthus nanus, a desert shrub
Source: Gigascience. 2018 Jun 28;7(7):giy074. doi: 10.1093/gigascience/giy074 (PMC6048559; doi:10.1093/gigascience/giy074)
Supplement: GIGA-D-17-00264_Revision_1.pdf [file giy074_giga-d-17-00264_revision_1.pdf]

GigaScience

Long-read sequencing and de novo genome assembly of Ammopiptanthus nanus, a desert shrub

--Manuscript Draft--

Manuscript Number:	GIGA-D-17-00264R1
Full Title:	Long-read sequencing and de novo genome assembly of Ammopiptanthus nanus, a desert shrub
Article Type:	Data Note
Funding Information:	the National Natural Science Foundation of China (31370356) — Prof. Yijun Zhou<br>the National Natural Science Foundation of China (31670335) — Dr. Fei Gao<br>the Ministry of Education of China through 111 projects (B08044) — Prof. Yijun Zhou<br>the Ministry of Education of China through 985 projects (YLDX01013) — Prof. Yijun Zhou
Abstract:	Background<br>Ammopiptanthus nanus is a rare broad-leaved shrub in the desert and arid regions of Central Asia. This plant species exhibits extremely high tolerance to drought and freezing and has been used in abiotic tolerance research in plants. As a relic of the Tertiary period, A. nanus is of great significance to plant biogeographic research in the ancient Mediterranean region. Here we report a draft genome assembly using the PacBio platform and gene annotation for A. nanus.<br>Findings<br>A total of 64.72 gigabases (Gb) of raw PacBio Sequel reads were generated from four 20 kb libraries. After filtering, 64.53 Gb of clean reads were obtained, giving 72.59 × coverage depth. Assembly using Canu gave an assembly length of 823 Mb, with a contig N50 of 2.76 Mb. The final size of the assembled A. nanus genome (823 Mb) was close to the 889 Mb estimated by k-mer analysis. The genome completeness was evaluated by BUSCO, and 1,328 out of the 1,440 conserved genes (92.22%) could be found in the A. nanus assembly. Genome annotation revealed that 74.09% of the A. nanus genome is composed of repetitive elements, 70.71 % of transposable elements, and 53.38 % of long terminal repeat elements (LTRs). We predicted 37,259 protein-coding genes, of which 96.7% were functionally annotated.<br>Conclusions<br>The genomic sequences of A. nanus could be a valuable source for comparative genomic analysis in the legume family, and will be useful for understanding the phylogenetic relationships of the Thermopsideae and the evolutionary response of plant species to the Qinghai Tibetan Plateau uplift.
Corresponding Author:	Fei Gao<br>Minzu University of China<br>Beijing, CHINA
Corresponding Author Secondary Information:	
Corresponding Author's Institution:	Minzu University of China
Corresponding Author's Secondary Institution:	
First Author:	Fei Gao
First Author Secondary Information:	
Order of Authors:	Fei Gao
	Xue Wang

Powered by Editorial Manager® and ProduXion Manager® from Aries Systems Corporation

	Xuming Li
	Mingyue Xu
	Huayun Li
	Merhaba Abla
	Huigai Sun
	Shanjun Wei
	Jinchao Feng
	Yijun Zhou
**Order of Authors Secondary Information:**	
**Response to Reviewers:**	Dear editor,<br><br>Enclosed you will find the manuscript GIGA-D-17-00264-R1, the revised version of the originally submitted paper entitled “Long-read sequencing and de novo genome assembly of Ammopiptanthus nanus, a desert shrub” that we are re-submitting for publication in GigaScience as Data Note.<br><br>Thank you for the opportunity to revise the manuscript. We have responded to all comments and suggestions made by the reviewers. Our responses to each comment are given below and changes in the revised manuscript are shown using track changes. We sincerely appreciate the reviewers’ comments that really helped us improving the manuscript and we hope that all the issues raised were addressed at satisfaction.<br><br>Thank you for your consideration. We look forward to hearing from you soon.<br><br><br>Regards<br>Fei Gao (on behalf of all co-authors)<br>College of life and environmental sciences,<br>Minzu University of China, Beijing, 100081, China<br>E-mail: gaofei@muc.edu.cn.<br>-----------------<br><br>Reviewer reports:<br><br>Reviewer #1: This report describes a standard, PacBio-based sequence and assembly for Ammonpiptanthus. The underlying sequencing technology is reasonable, apparently involving DNA template of acceptable length and quality and leading to sequence reads and depth suitable for whole genome assembly. Subsequent analyses (k-mer, repeat, genome feature annotation) are standard with methods described in sufficient detail. Accompanying files are typical and sufficient for most follow-up analyses researchers may pursue. However, there are numerous English grammar errors in the text that should be corrected.<br>Response：The manuscript has been improved with the help of an English editing service, Editage.<br><br>Citation(s) supporting the statement that the genus is interesting and characterized in terms of abiotic stress tolerance are missing (that is, plant physiology research, not just transcriptomic analyses).<br>Response：We have added three additional plant physiology research related literatures in the reference section.<br><br>Non-genomic research supporting the very low level of heterozygosity, should accompany the value reported here. Finally, locations in the text where accompanying data files are available should be highlighted within the text.<br>Response：We have deposited the related data according to Gigascience's requirement. And we have added the NCBI SRA accession numbers of the related data in the corresponding locations in the text.

Powered by Editorial Manager® and ProduXion Manager® from Aries Systems Corporation

Reviewer #2:
This manuscript presented the genome assembly and annotation of Ammopiptanthus nanus. This dataset could add more resources for plant genomics, especially for the dry land plant research. Below are my detailed concerns:

1) As a genomics resource, the authors didn't release the data properly. Even though the raw sequencing reads are in SRA, a lot of future data users would look for the genome assembly and annotation. A genome browser and ftp will be requested. Reference genome would request further improvement and maintenance. I would suggest the authors to have a genome browser up or deposit the genome to the large databases like NCBI, Ensembl-plants. In this way, users would have better access to the genome. On the hand, the feedback from the users could help to improve this geneome.
Response： We have deposited the related data according to Gigascience's requirement.

2) As a manuscript it is not well organized. There are seven tables, five of which only have one row except for the header. These numbers have been described in the text. These tables should be reorganized.
Response：Thank you for your suggestion. The five tables with one row were deleted (Table 1), or moved to the supplementary material (Table 2, 4, 6, 7 and 9).

3) It doesn't make a lot of sense to have a main figure to only show the look of the plant. As the author tried to introduce this species, an evolutionary species tree would be more informative.
Response：The picture of Ammopiptanthus nanus will be kept according to editor’s advice.

4) The last paragraph about the background (Line 61-63): "Most of the de novo assemblies of plant genomes reported recently have been performed using the next generation sequencing technologies such as Illumina or 454 sequencing platforms" is too subjective, as the authors would like to highlight their genome was done by PacBio. Recently there is also a list of complicated plant genomes being done by third generation sequencing: maize, sunflower, Oropetium thomaeum, rice, Chenopodium quinoa, thomaeum. Authors should not ignore these high genomes. They should be included in introduction.
Response：We have listed several plant species whose genomes were recently sequenced using third PacBio sequencing platform in introduction.

5) After the genome assembly, the contigs were only polished by Pilon using short reads. I would also recommend to also use PacBio reads to correct the base calling, as illumina reads may not be able to cover all the contigs due to alignment and sequencing bias. The number of bases were checked and corrected should be mentioned, too.
Response：When we use Pacbio or Illumina data to correct a genome, the same strategy is applied: reads were mapped to the genome and SNPs & INDELs were corrected. But after quiver correction, a lot of errors were still in the genome as the error ratio of the subreads is high. For example, Pacbio data was used to corrected the gorilla genome in the first step. After this step the QV of the genome was 30. Finally, Illumina data was used to corrected the genome and the author mentioned that “After error correction, we estimate that Susie3 has less than one error per 5000 bp (QV > 35)” (David Gordon, 2016).

In total, 56 Gb Illumina data (~70 fold coverage) was used to correct the final genome of A. nanus by multi-rounds. The data covered 99% of the assembled genome. The data is huge enough to cover all the genome and the depth is high.

And in the newest Pilon version (https://github.com/broadinstitute/pilon/releases) (the version was used to polish the A. nanus assembly in our study), the parameter “-fix bases” were supplied to correct both SNPs and INDELs. Furthermore, in the statistics of the final assembly, the number of SNPs and INDELs were very low. The table for error state of the genome is provided as below. In addition, all the genome assessment result showed that the final genome quality is good. In summary, we think our flowchart can achieve a good assembly without quiver or arrow correction.

Powered by Editorial Manager® and ProduXion Manager® from Aries Systems Corporation

Table 1 The error rate of the A. nanus genome after Pilon correction
Roundsnp_numinsert_numdelete_numerror_ratio (%)
Round199,23925,11883,3850.03
Round22,7341,7364,6370.00

6) Authors deployed several approaches for gene annotation. Figure 2 is not very informative. A flowchart figure describing how the gene annotation was done could be better. I also have concerns that only default parameters from the gene predictors were used for ab initio prediction. Many of these default prediction parameters were not suitable for plants. They request training models to generate accurate gene models. A comparative analysis with close related species will be strong evidence to show the quality of gene models. With the current data, there is no evaluation of the quality of gene models.
Response：Figure 2 has been replaced by a new Venn diagram plotted using UpSetR (Figure S2), and the new figure clearly depicted the integration of gene predictions using the three approaches.

Augustus, Genscan, GlimmerHMM, GeneID, and SNAP were used to conduct the ab initio gene prediction and all the original gene model was set to use the Arabidopsis gene model as the training models, a gene model almost used in gene model prediction of all plants. For Augustus' prediction, we took the Arabidopsis's gene model as the ab initial gene model, but also, the PASA's gene model was used as initial gene model for training. Finally, the best gene model with higher precision and specificity was selected as the gene model. At last the Ab initio-based, homolog-based and transcriptomic-based genes were integrated together using EVM. Finally, models with EVM score value more than 1000, length >=300 bp and the length of full CDS is the 3 times of integers were considered as genes.

We used the transcriptome data to assess the quality of the gene models by mapping the data to the whole genome using Tophat (Table S5).

Reviewer #3:
The article (Manuscript#: GIGA-D-17-00264) entitled "Long-read sequencing and de novo genome assembly of Ammopiptanthus nanus, a desert shrub", by Fei Gao and collaborators delivers "a de novo assembly of the rare broad-leaved shrub Ammopiptanthus nanus". The work presents the completeness of the assembled genome as well its gene and repeat annotation. It's major finding is the high amonunt of repetitive elements and the authors suggest their work as a valuable source for comparative genomics analysis in the family of legumes. I agree with most of what the authors state but have the following suggestions:

1) The size of the genome was estimated via k-mer distribution. Figure S1 shows a major and a minor peak. I agree with the authors that the highest peak represents the diploid genome given the low amounts of BUSCO duplicates. Nevertheless, a in-depth description of the repetitve peak (better contigs associated with it) would be interesting and should be added to the manuscript. Please describe how much contigs and how much total sequence are duplicated (showing k-mers from the second peak) and elaborate on the annotated genes/repeats you can find of there. Consider moving the k-mer histogram and maybe a graphical (GO enrichment based) summary of duplicated genes/repeats to the main text as figure.
Response：Thank you for your kind advices. As we know, few genes were located at the repeat region and we predicted the gene model using the masked genome. The kind of repeat elements and their contents were obtained in the repeat annotation step and supplied in table S4. Indeed, there exists genes with multi-copies but few genes with high copies might be counted and appeared at the second peak when their sequences were cut to be K-mers as the length of their sequences were quite small compared to the whole genome.

2) The genome was assembled with Canu and polished with Pilon. Could the authors explain while the initial Canu assembly was not polished with Arrow prior to Pilon polishing. A lot of medium sized InDels usually remain in the assembly if only Pilon is used for assembly polishing. The final genome release should include a Arrow polishing step. The genome assembly should be deposited in one of the public

Powered by Editorial Manager® and ProduXion Manager® from Aries Systems Corporation

	databases.<br>Response: Please see our response to the questions 5 of the review2.<br>The genome assembly was deposited in GigaDB according to Gigascience's requirement.<br><br>3) Repeats and genes were predicted with various tools. The text is missing details on which models the ab initios gene predictors were used with (e.g., Augustus, SNAP). Please describe how these models were generated.<br>Response: The Ab initio gene predictors include Augustus, GeneID, Genescan, GlimmerHMM and SNAP. All these softwares were trained using the Arabidopsis gene model before gene prediction. For Augustus, PASA's gene model was also used as initial gene model for training.<br>The following descriptions have been added in our manuscript:<br>“and all these software packages were trained using the Arabidopsis gene model before gene prediction. For gene prediction using Augustus, besides the Arabidopsis's gene model, the PASA's gene model was also used as initial gene model for training. Finally, the best gene model with higher accuracy and specificity was used. Quality evaluation of gene models was conducted by aligning transcriptome sequences to the whole genome assembly using Tophat (Table S5).”.<br><br>4) Line 145-148: Please remove the term "functionally annotated" and repleace it with something appropriate (e.g., classified into families and predicting domains and important sites). The tools mentioned are annotating evolutionary conserved domains or lift over putative functions via homology-based methods but none of them are able to annotate genes functionally.<br>Response: The term "functionally annotated" was replaced by “classified into families according to their putative functions”.<br><br>5) Please add a short section/sentence about the gene models that were choosen by EVM. Simply indicate which was the prefered gene prediction tool/model that EVM selected from.<br>Response: The following descriptions have been added in our manuscript.<br><br>“Higher weights were assigned to the PASA predicted transcripts from unigenes and GeMoMa predicted homologous transcripts than to the ab initio predicted transcripts when conducting the EVM integration.”<br><br>6) Please indicate whether default mapping parameters were used to assess the genome completeness via short read mappings and adopt the quality assessment method from (Bickhart et al. 2017, Nat Genet.; Jain et al. 2017, bioRxiv).<br>Response: Default mapping parameters were used to assess the genome completeness via Illumina short read mappings. We adopted a similar assessment method from (Bickhart et al. 2017, Nat Genet.) to assess the genome completeness via short read mappings. We used BWA (0.7.10-r789) to map the Illumina data to the genome using default parameters. The Q30 of our data is 91.67% and the properly mapping rate is up to 98%. We also used BUSCO and EST to assess the genome and the scores are 92.22% and 100% respectively. All assessment results indicated that the completeness of our genome is pretty high.<br><br>7) Please indicate that the data used for Pilon polishing is the same that is used during the completeness assessment and state any eventual bias.<br>Response: The same Illumina data were used to proceed the Pilon polishing and genome completeness assessment. The bias details were supplied in Table S8.<br><br>8) Please replace Figure 2 with either a scaled Venn diagram but better with a UpSetR plot to ease interpretation of the gene prediction integration. Further consider moving the Figure 2 to the supplement since Table 3 is more than enough.<br>Response: Thank you for your advice. We replaced Figure 2 with a new Venn diagram plotted using UpSetR, and the new figure were moved to supplementary file as Figure S2.
**Additional Information:**	
**Question**	**Response**

Powered by Editorial Manager® and ProduXion Manager® from Aries Systems Corporation

Are you submitting this manuscript to a special series or article collection?	No
**Experimental design and statistics**<br><br>Full details of the experimental design and statistical methods used should be given in the Methods section, as detailed in our Minimum Standards Reporting Checklist. Information essential to interpreting the data presented should be made available in the figure legends.<br><br>Have you included all the information requested in your manuscript?	Yes
**Resources**<br><br>A description of all resources used, including antibodies, cell lines, animals and software tools, with enough information to allow them to be uniquely identified, should be included in the Methods section. Authors are strongly encouraged to cite Research Resource Identifiers (RRIDs) for antibodies, model organisms and tools, where possible.<br><br>Have you included the information requested as detailed in our Minimum Standards Reporting Checklist?	Yes
**Availability of data and materials**<br><br>All datasets and code on which the conclusions of the paper rely must be either included in your submission or deposited in publicly available repositories (where available and ethically appropriate), referencing such data using a unique identifier in the references and in the "Availability of Data and Materials" section of your manuscript.<br><br>Have you have met the above requirement as detailed in our Minimum Standards Reporting Checklist?	Yes

Manuscript

Click here to download Manuscript De novo genome assembly of Ammopiptanthus nanus_revised_1.docx

Long-read sequencing and *de novo* genome assembly of *Ammopiptanthus nanus*, a desert shrub

Fei Gao[1], Xue Wang[1], Xuming Li[2], Mingyue Xu[2], Huayun Li[3], ~~Abla~~ Merhaba Abla[1], Huigai Sun[1], Shanjun Wei[1], Jinchao Feng[1*], Yijun Zhou[1*]

[1]College of Life and Environmental Sciences, Minzu University of China, Beijing, 100081, China

[2]Biomarker Technologies Corporation, Beijing, 101300, China.

[3]Annoroad Genomics, Beijing, 100176, China

Email addresses: Fei Gao <gaofei@muc.edu.ecn>, Xue Wang <wangxue@muc.edu.cn>, Xuming Li<lixm@biomarker.com.cn>, Mingyue Xu<xumy@biomarker.com.cn>, Huayun Li < huayunli@annoroad.com >, Abla Merhaba< Merhaba@muc.edu.cn >, Huigai Sun <sunhuigai66@163.com>, Shanjun Wei<wei.s.j@163.com>

*Correspondence should be addressed to: Y. Z. <zhouyijun@muc.edu.cn>, J. F. <fengjinchao@muc.edu.cn>

Abstract

Background

Ammopiptanthus nanus is a rare broad-leaved shrub in the desert and arid regions of Central Asia. This plant species exhibits extremely high tolerance to drought and freezing ~~stresses~~ and has been used in abiotic tolerance research in plants. As a relic of the Tertiary period, *A. nanus* is of great significance to plant biogeographic research in the ancient Mediterranean region. Here we report a draft genome assembly using the PacBio platform and gene annotation for *A. nanus*.

Findings

A total of 64.72 gigabases (Gb) of raw PacBio Sequel reads were generated from four 20 kb libraries. After filtering, 64.53 Gb of clean reads were obtained, giving 72.59 × coverage depth. Assembly using Canu gave an assembly length of 823 Mb, with a contig N50 of 2.76 Mb. The final size of the assembled *A. nanus* genome (823 Mb) was close to the 889 Mb estimated by k-mer analysis. The genome completeness was evaluated by BUSCO, and 1,328 out of the 1,440 conserved genes (92.22%) could be found in the *A.*

nanus assembly. Genome annotation revealed that 74.09% of the *A. nanus* genome is composed of repetitive elements, 70.71 % of transposable elements, and 53.38 % of long terminal repeat elements (LTRs). We predicted 37,259 protein-coding genes, of which 96.7% were functionally annotated.

Conclusions

The genomic sequences of *A. nanus* could ~~provide~~ be a valuable source for comparative genomic~~s~~ analysis in the legume family ~~legume~~, and will be useful for understanding the phylogenetic relationships of the Thermopsideae and the evolutionary response of plant species to the Qinghai Tibetan Plateau uplift.

Keywords

Ammopiptanthus nanus, PacBio sequencing, Genome assembly, Genome annotation

Data Description

Background information

Ammopiptanthus nanus, a desert shrub and a relic from the tertiary period, is one of two species in the genus *Ammopiptanthus*. ~~and~~ T~~t~~his genus belongs to the tribe Thermopsideae and the~~,~~ family Fabaceae (Figure 1). *Ammopiptanthus* is the only genus of evergreen broadleaf shrub distributed in the desert and arid regions of Central Asia, and the plants in this genus play important ecological roles by fixing moving sands and delaying further desertification [1].

Tribe Thermopsideae is considered to be a basal branch in the family Fabaceae and the habitats of the ca. 45 plant species in t~~T~~ribe Thermopsideae are interspersed among the Mediterranean Basin, C~~c~~entral Asia, and temperate North America. Studies on the molecular biology of these plant species will promote understanding of the phylogeny of family Fabaceae, as well as ~~and of~~ some interesting biogeographical topics, such as how the Qinghai-Tibetan Plateau uplift and Tethys retreat affected plant evolution [2, 3]. In addition, the genus *Ammopiptanthus* is a unique and isolated branch in tribe Thermopsideae~~Thermopsidea~~. There are still some debates about the evolution and phylogeny of this genus [3], and more molecular evidence is needed to clarify ~~those~~ these issues.

~~The plant S~~Species in g~~G~~enus *Ammopiptanthus* exhibit extremely high tolerance to drought and freezing ~~stresses~~ and ~~has~~ have been used in abiotic tolerance research in plants [4–6]. Although several transcriptome analyses~~is~~ of the response to drought and cold stress have been conducted [1, ~~4~~7–~~6~~9], the lack of genome sequence information impedes ~~the~~ further investigation into the molecular mechanism underlying the stress tolerance of *Ammopiptanthus* species.

Most of the *de novo* assemblies of plant genomes recently reported ~~recently~~ have been performed using the next generation sequencing technologies such as Illumina or 454 sequencing platforms [~~7~~10–~~9~~12]. However, ~~those~~ these assemblies generally have ~~a~~ low N50 values and a large number of contigs, partly because of the complexity of the plant genome. The newly developed Pacific BioSciences (PacBio) sequencing platform, a third-generation sequencing technology, has started to address some of the intrinsic challenges in sequencing and assembling large and complex plant genomes, via producing tens of thousands of long individual reads (up to ~40 kb) [~~10~~13]. Recently, several complicated plant genomes, including those of maize [14], sunflower [15], and *Chenopodium quinoa* [16], have been sequenced using the PacBio sequencing technology. In the present study, we employed single molecule real-time (SMRT) sequencing developed by PacBio, to generate a draft genome assembly for *A. nanus*.

Formatted: Font: Italic

Sample collection and genomic DNA sequencing

The leaf tissues of a single *A. nanus* tree (NCBI Taxonomy ID: 111851) were collected from Xinjiang, China. After collection, tissues were immediately transferred into liquid nitrogen and stored until DNA extraction. The extraction of DNA was conducted using the CTAB method according to the protocol 'Preparing *Arabidopsis* Genomic DNA for Size-Selected ~20 kb SMRTbell™ Libraries' (http://www.pacb.com/wp-content/uploads/2015/09/Shared-Protocol-Preparing-Arabidopsis-DNA-for-20-kb-SMRTbell-Libraries.pdf). The quality of the extracted genomic DNA was checked by 1% agarose gel electrophoresis, and the concentration was quantified using a Qubit fluorimeter (Invitrogen, Carlsbad, CA, USA).

Formatted: Font: Italic

Long-read sequencing was performed at Biomarker Technologies Corporation (Beijing, China) with a PacBio Sequel sequencer (Pacific Biosciences, Menlo Park, CA, USA). The SMRT Bell library was prepared using a DNA Template Prep Kit 1.0 (PacBio p/n 100-259-100) and four 20 kb SMRTbell libraries were constructed. Genomic DNA (10 µg) was mechanically sheared using a Covaris g-Tube (Kbiosciences p/n 520079) with a goal of~~aiming at~~ DNA fragments of approximately~~about~~ 20 kb. A Bioanalyzer 2100 12K DNA Chip assay (Agilent p/n 5067-1508) was used to assess the fragment size distribution. Sheared genomic DNA (5 µg) was DNA-damage repaired and end-repaired using polishing enzymes. A blunt-end ligation reaction followed by exonuclease treatment was conducted to generate the SMRT Bell template. A Blue Pippin device (Sage Science, Inc., Beverly, MA, USA) was used to size select the SMRT Bell template and enrich large fragments (> 10 kb). The size-selected library was quality inspected and quantified on an Agilent Bioanalyzer 12 kb DNA Chip (Agilent Technologies, Santa Clara, CA, USA) and a Qubit f~~F~~luorimeter (Invitrogen, Carlsbad, CA, USA)~~(Life Technologies)~~. A ready-to-sequence SMRT Bell-Polymerase Complex was created using a Binding Kit 2.0 (PacBio p/n 100-862-200), according to the manufacturer's instructions. The Sequel instrument was programmed to load and sequence the sample on PacBio SMRT cells v3.0 (PacBio p/n 100-171-800), acquiring one movie of 360 min per SMRT cell. The MagBead loading (PacBio p/n 100-125-900) method was employed ~~in order~~ to improve the enrichment of the larger fragments. A total of 13 SMRT cells were processed yielding 64.72 G subread sequences.

For Illunima sequencing, paired-end libraries with insert sizes of 350 bp were constructed with the standard protocol provided by Illumina (San Diego, CA, USA) and sequenced on an Illumina HiSeq X ten platform. A total of 55.97 Gb of paired-end (2 × 150 bp) clean sequences were generated (Table S1). These data were used for genome size estimation, correction of genome assembly, and assembly evaluation.

Genome size estimation

We characterized the genome size and heterozygosity using the distribution of k-mers of length 19 from the ~~Illumine~~ Illumina HiS~~s~~eq reads (55.97 Gb clean reads from 350 bp insert size library, NCBI SRA accession number: SRX3286209). This analysis was performed using "kmer_freq_stat" software (developed by Biomarker Technologies). The genome size (G) of *A. nanus* was estimated by the following formula: G =

k-mer number/average k-mer depth, ~~here,~~where k-mer number = t~~T~~otal k-mers - abnormal k-mers (with too low or too high frequency). The highest peak in ~~at~~ the k-mer distribution curve was found at the k-mer depth of 53, with a k-mer number of 47,408,863,457 (Figure S1). The peak at depth of more than 106 was a repetitive peak (k-mers duplicated ~~due to~~because of repetition). Finally, the *A. nanus* genome size was estimated to be 888.92 Mb, the heterozygosity ~~is~~ was approximately 0.02%, and the data used in 19-mer analysis was approximately~~about~~ 53× coverage of the genome.

Genome assembly

The Sequel raw bam files were converted into subreads in fasta format using the standard PacBio SMRT software package (read data is available at the NCBI SRA accession number: SRX3262947). Then subreads of ~~shorter~~ less than 500 bp were filtered out. Finally, 7,918,322 reads and 64,538,018,400 bases (~ 73 × depth) were produced. The average subread length was 8.15 kb with a N50 length of 12.79 kb (Table ~~1 and~~ S2). The genome assembly was conducted using Canu software (v1.5) [1~~1~~7] (correctedErrorRate=0.045, corOutCoverage=70). Then, the resulting draft genome was polished by Pilon (v1.22, RRID:SCR_014731) [~~12~~18] using the default settings to correct sequencing errors in genome assembly. Finally, we assembled a genome of 823 Mb with 1,099 contigs and contig N50 of 2.76 Mb (Table ~~2~~S3).

Repeat annotation and gene prediction

For repeat detection, first, four software packages, i.e., LTR-FINDER (v1.0.5) [~~13~~19], MITE-Hunter (v1.0.0) [20~~14~~], PILER (v1.0) [21~~15~~], and RepeatScout (v1.0.5, RRID:SCR_014653) [22~~16~~] were used to build a *de novo* repeat library on the basis of our assembly with the default settings, and then, the predicted repeats were classified using PASTEClassifier (v1.0) [~~17~~23] and merged with Repbase (19.06) [24~~18~~].~~,~~ F~~f~~inally, using the resulting repeat database as the final repeat library, RepeatMasker (v4.0.5; RepeatMasker, RRID:SCR 012954) [~~19~~25] was utilized to identify repetitive sequences in the *A. nanus* genome with the following parameters "-nolow -no_is -norna -engine wublast". Overall, approximately 610.22 Mb of repetitive sequences (74.09% of the assembly) were detected, containing 439.66 Mb (53.38% of the assembly) LTRs (Table ~~S3~~S4).

Ab initio~~-~~based, homolog~~-~~based, and RNA-seq~~-~~based gene prediction methods were conducted in combination to identify the protein-coding genes in the *A. nanus* genome assembly. Genscan [~~20~~26], Augustus (v2.4, RRID:SCR_008417) [~~21~~27], GlimmerHMM (v3.0.4, RRID:SCR_002654) [16], GeneID (v1.4) [~~22~~28], and SNAP (v2006-07-28, RRID:SCR_002127) [~~23~~29] with the default parameters were employed for the *Ab initio* based gene prediction, and all these software packages were trained using the *Arabidopsis* gene model before gene prediction. For gene prediction using Augustus, besides the Arabidopsis's gene model, the PASA's gene model was also used as initial gene model for training. Finally, the best gene model with higher accuracy and specificity was used. Quality evaluation of gene models was conducted by aligning transcriptome sequences to the whole genome assembly using Tophat (Table S5). GeMoMa (v1.3.1) [~~24~~30] was used in homolog~~-~~based gene annotation and the protein database of *Cicer arietinum* (GCA_000331145.1), *Phaseolus vulgaris* (GCA_000499845.1), *Glycine max* (GCA_000004515.3), and *Arachis duranensis* (GCA_000817695.2) from GenBank were used as the reference databases. For the RNA-seq~~-~~based method of gene prediction, TransDecoder (v2.0, http://transdecoder.github.io), GeneMarkS-T (v5.1, RRID:SCR_011930) [31~~25~~], and PASA (v2.0.2, RRID:SCR_014656) [~~26~~32] were used, and the *A. nanus* transcriptome data were assembled in a previous study (NCBI SRA accession number: SRX1409432 and SRX1406652) [33]. Finally, the results from the three methods were integrated using EVM (v1.1.1, RRID:SCR_014659) [~~27~~34]. Higher weights were assigned to the PASA predicted transcripts from unigenes and GeMoMa predicted homologous transcripts than to the *ab initio* predicted transcripts when conducting the EVM integration. ~~Totally~~In total, a gene set with 37,259 protein-coding genes was predicted from the *A. nanus* genome assembly (Table 1~~3~~ and ~~4~~S6, and Figure S2). These genes were scattered over 1,099 contigs, averaging 33.90 genes per contig. By alignment to the NR, Nt, KOG [~~28~~35], GO [~~29~~36], KEGG (RRID:SCR_001120) [30], Swissprot (RRID:SCR_002380) [~~31~~37], TrEMBL [~~31~~38], and Pfam databases (RRID:SCR_004726) [~~32~~39] using blast with an e-value cutoff of 1E-5, 96.70% of the predicted genes could be classified into families according to their putative functions~~functionally annotated~~ (Table ~~5~~2).

For pseudogene prediction, first, GenBlastA [40~~33~~] was used to scan the *A. nanus* genome for sequences homologous to the known protein-coding genes it contain<u>ed</u>~~s~~, then GeneWise (RRID:SCR_015054) [<u>41</u>~~34~~] was adopted to search the premature stop codon<u>s</u> or frameshift mutation<u>s</u> in those sequences and, consequently, to identify pseudogenes. In total, 7,588 pseudogenes were identified from the *A. nanus* genome (Table <u>S7</u>~~6~~).

Completeness of the genome assembly

First, the 55.97G Illunima sequencing reads <u>(NCBI SRA accession number: SRX3286209)</u> used for k-mer analysis were aligned to the *A. nanus* genome assembly using bowtie [<u>42</u>~~35~~]. The result<u>s</u> showed that all Illunima reads were mapped and 98.07% PE reads were mapped concordantly (Table <u>S8</u>~~7~~).

Second, the *A. nanus* unigenes assembled in a previous study <u>(NCBI SRA accession number: SRX1409432 and SRX1406652) [33]</u> were aligned to the *A. nanus* genome using BLAT v0.36 (BLAT, RRID:SCR_011919) [<u>43</u>~~36~~] with default parameters. The alignment indicated that 100% of unigenes (> 500 bp in length) assemblies <u>were</u> mapped to the *A. nanus* genome assembly (Table ~~8~~<u>3</u>).

We also evaluated the completeness of the genome assembly of *A. nanus* by using BUSCO v2.0 (BUSCO, RRID:SCR_015008) [<u>44</u>~~37~~]. The results showed that 92.22% (1,328 out of 1,440 BUSCOs) of plant~~s~~ set<u>s</u> (embryophyta_odb9, download from http://busco.ezlab.org/) were identified as complete in the *A. nanus* assembly (Table ~~9~~<u>S9</u>). Together, the results indicated that our dataset represented a genome assembly with a high level of coverage.

Conclusion

In summary, the draft genome sequence of *A. nanus* obtained in the present study <u>demonstrated</u>~~shows~~ that third-generation sequencing technology~~-~~<u>, such as</u>~~like~~ <u>the</u> PacBio platform<u>,</u> could be useful in deciphering complex plant genomes. The availability of the *A. nanus* genome sequence should facilitate *de novo* genome assembly of other species in this genus. The datasets from the present manuscript could not only provide <u>a</u> valuable source for further comparative genomics analysis in <u>the legume</u> family~~ legume,~~ <u>and</u>

help to answer some important questions related to the biogeography research in the ancient Mediterranean region, but also facilitate understanding of how plants adapt to the stressful conditions in temperate deserts in Central Asia.

List of abbreviations

Gb: Giga base; TE: Transposable element; GO: Gene Ontology; PE: paired-end; LTR: Long terminal repeat element; CDS: Coding DNA sequence; SMRT: Single molecule real-time; PacBio: Pacific BioSciences; KOG: Eukaryotic Orthologous Groups of proteins; KEGG: Kyoto Encyclopedia of Genes and Genomes

Competing interests

The authors declare that they have no competing interests.

Funding

This work was financially supported by the National Natural Science Foundation of China (31370356 and 31670335) and the Ministry of Education of China through 111 and 985 projects (B08044, YLDX01013).

Availability of Supporting Information

Raw genomic sequence reads are available in the NCBI Sequence Read Archive under project number PRJNA413722. Supporting data are also available from the GigaScience database (GigaDB) [3845].

Author Contributions

Y.Z and J.F oversaw the project. M.A., M. X.W. and S.W. collected the sample and extracted the genomic DNA. X.L., H.L., M.X., and H.S performed the genome assembly, annotated the genome and analyzed data. F.G analyzed data. F.G and Y.Z wrote the manuscript.

References

1. Gao F, Wang J, Wei S, Li Z, Wang N, Li H. Transcriptomic analysis of drought stress responses in *Ammopiptanthus mongolicus* leaves using the RNA-Seq technique. Plos ONE. 2015;10(4):e0124382.
2. Zhang ML, Huang JF, Sanderson SC, Yan P, Wu YH, Pan BR. Molecular biogeography of tribe Thermopsideae (Leguminosae): A Madrean-Tethyan disjunction pattern with an African origin of core Genistoides. Biomed Res Int. 2015; 2015:864804.
3. Shi W, Liu PL, Duan L, Pan BR, Su ZH. Evolutionary response to the Qinghai-Tibetan Plateau uplift: phylogeny and biogeography of *Ammopiptanthus* and tribe Thermopsideae (Fabaceae). Peer J. 2017;5:e3607.
4. Xu S, An L, Feng H, Wang X, Li X. The seasonal effects of water stress on *Ammopiptanthus mongolicus* in a desert environment. J Arid Environ. 2002; 51(3):437–47.
5. Wang W, Chen Y, Liu M, Lu C. Effects of cold-hardening on compatible solutes and antioxidant enzyme activities related to freezing tolerance in *Ammopiptanthus mongolicus* seedlings. For Stud China. 2008;10(2):101–6.
~~3.~~6. Gao T-P, Chen T, Feng H-Y, An L-Z, Xu S-J, Wang X-L. Seasonal and annual variation of osmotic solute and stable carbon isotope composition in leaves of endangered desert evergreen shrub *Ammopiptanthus mongolicus*. S Afr J Bot. 2006; 72(4):570–8.
~~4.~~7. Zhou Y, Gao F, Liu R, Feng J, Li H. *De novo* sequencing and analysis of root transcriptome using 454 pyrosequencing to discover putative genes associated with drought tolerance in *Ammopiptanthus mongolicus*. BMC Genomics. 2012;13:266.
~~5.~~8. Wu Y, Wei W, Pang X, Wang X, Zhang H, Dong B, et al. Comparative transcriptome profiling of a desert evergreen shrub, *Ammopiptanthus mongolicus*, in response to drought and cold stresses. BMC Genomics. 2014;15:671.
~~6.~~9. Pang T, Ye CY, Xia X, Yin W. *De novo* sequencing and transcriptome analysis of the desert shrub, *Ammopiptanthus mongolicus*, during cold acclimation using Illumina/Solexa. BMC Genomics. 2013;14:488.
~~7.~~10. Fu Y, Li L, Hao S, Guan R, Fan G, Shi C, et al. Draft genome sequence of the Tibetan medicinal herb Rhodiola crenulata. Gigascience. 2017;6(6):1–5.

Formatted: Indent: Left: 0", Hanging: 0.3"

~~8.~~11. Zhao D, Hamilton JP, Pham GM, Crisovan E, Wiegert-Rininger K, Vaillancourt B, et al. *De novo* genome assembly of *Camptotheca acuminata*, a natural source of the anti-cancer compound camptothecin. Gigascience. 2017;6(9):1–7.

~~9.~~12. Xia EH, Zhang HB, Sheng J, Li K, Zhang QJ, Kim C, et al. The tea tree genome provides insights into tea flavor and independent evolution of caffeine biosynthesis. Mol Plant. 2017;10(6):866–77.

13. Roberts RJ, Carneiro MO, Schatz MC. The advantages of SMRT sequencing. Genome Biol. 2013;14(7):405.

14. Jiao Y, Peluso P, Shi J, Liang T, Stitzer MC, Wang B, et al. Improved maize reference genome with single-molecule technologies. Nature. 2017;546(7659):524–7.

15. Badouin H, Gouzy J, Grassa CJ, Murat F, Staton SE, Cottret L, et al. The sunflower genome provides insights into oil metabolism, flowering and Asterid evolution. Nature. 2017;546(7656):148–52.

~~10.~~16. Jarvis DE, Ho YS, Lightfoot DJ, Schmöckel SM, Li B, Borm TJ, et al. The genome of *Chenopodium quinoa*. Nature. 2017;542(7641):307–12.

~~11.~~17. Koren S, Walenz BP, Berlin K, Miller JR, Bergman NH, Phillippy AM. Canu: scalable and accurate long-read assembly via adaptive k-mer weighting and repeat separation. Genome Res. 2017;27(5):722–36.

~~12.~~18. Walker BJ, Abeel T, Shea T, Priest M, Abouelliel A, Sakthikumar S, et al. Pilon: an integrated tool for comprehensive microbial variant detection and genome assembly improvement. PLoS ONE. 2014;9(11):e112963.

~~13.~~19. Xu Z, Wang H. LTR˙FINDER: an efficient tool for the prediction of full-length LTR retrotransposons. Nucleic Acids Res 2007;35(web server issue):W265–8.

~~14.~~20. Han Y, Wessler SR. MITE-Hunter: a program for discovering miniature inverted-repeat transposable elements from genomic sequences. Nucleic Acids Res. 2010;38(22):e199.

~~15.~~21. Edgar RC, Myers EW. PILER: identification and classification of genomic repeats. Bioinformatics. 2005;21:i152–8.

~~16.~~22. Price AL, Jones NC, Pevzner PA. *De novo* identification of repeat families in large genomes. Bioinformatics 2005;21 (suppl 1):i351–8.

~~17.~~23. Hoede C, Arnoux S, Moisset M, Chaumier T, Inizan O, Jamilloux V, et al. PASTEC: An automatic transposable element classification tool. PLoS ONE. 2014;9:e91929.

~~18.~~24. Bao W, Kojima KK, Kohany O. Repbase Update, a database of repetitive elements in eukaryotic genomes. Mobile DNA. 2015;6:11.

~~19.~~25. Tarailo-Graovac M, Chen N. Using RepeatMasker to identify repetitive elements in genomic sequences. Curr Protoc Bioinformatics. 2009; Chapter 4: Unit 4.10.

~~20.~~26. Burge C, Karlin S. Prediction of complete gene structures in human genomic DNA. J Mol Biol. 1997; 268:78–94.

~~21.~~27. Stanke M, Waack S. Gene prediction with a hidden Markov model and a new intron submodel. Bioinformatics. 2003;19 Suppl 2:ii215–25.

~~22.~~28. Blanco E, Parra G, Guigó R: Using geneid to identify genes. Curr Protoc Bioinformatics. 2007;4.3.

~~23.~~29. Korf I. Gene finding in novel genomes. BMC bioinformatics. 2004;5:59.

~~24.~~30. Keilwagen J, Wenk M, Erickson JL, Schattat, MH, Jan, G, Frank, H. Using intron position conservation for homology-based gene prediction. Nucleic Acids Res. 2016;44:e89.

~~25.~~31. Tang S, Lomsadze A, Borodovsky M. Identification of protein coding regions in RNA transcripts. Nucleic Acids Res. 2015;43(12):e78.

32. Campbell MA, Haas BJ, Hamilton JP, Mount SM, Buell CR. Comprehensive analysis of alternative splicing in rice and comparative analyses with *Arabidopsis*. BMC genomics. 2006;7:327.

~~26.~~33. Gao F, Li H, Xiao Z, Wei C, Feng J, Zhou Y, *De novo* transcriptome analysis of *Ammopiptanthus nanus* and its comparative analysis with *A. mongolicus*. Trees. 2017; DOI: 10.1007/s00468-017-1631-6.

~~27.~~34. Haas BJ, Salzberg SL, Zhu W, Pertea M, Allen JE, Orvis J, et al. Automated eukaryotic gene structure annotation using EVidenceModeler and the Program to Assemble Spliced Alignments. Genome Biol. 2008;9(1):R7.

~~28.~~35. Tatusov RL, Natale DA, Garkavtsev IV, Tatusova TA, Shankavaram UT, Rao BS, et al. The COG database: new developments in phylogenetic classification of proteins from complete genomes. Nucleic Acids Res. 2001;29(1):22–8.

~~29.~~36. Dimmer EC, Huntley RP, Alam-Faruque Y, Sawford T, O'Donovan C, Martin MJ, et al. The UniProt-GO annotation database in 2011. Nucleic Acids Res. 2012:40(Database issue):D565–70.

~~30.~~37. Kanehisa M, Goto S. KEGG: Kyoto Encyclopedia of Genes and Genomes. Nucleic Acids Res. 2000;28(1):27–30.

~~31.~~38. Boeckmann B, Bairoch A, Apweiler R, Blatter M-C, Estreicher A, Gasteiger E, et al. The SWISS-PROT protein knowledgebase and its supplement TrEMBL in 2003. Nucleic Acids Res. 2003;31(1):365–70.

~~32.~~39. Zdobnov EM, Apweiler R. InterProScan–an integration platform for the signature-recognition methods in InterPro. Bioinformatics. 2001;17:847–8.

~~33.~~40. She R, Chu JS, Wang K, Pei J, Chen N. GenBlastA: enabling BLAST to identify homologous gene sequences. Genome Res. 2009;19(1):143–9.

~~34.~~41. Birney E, Clamp M, Durbin R. GeneWise and genomewise. Genome Res. 2004;14(5):988–95.

~~35.~~42. Langmead B, Trapnell C, Pop M, Salzberg SL. Ultrafast and memory-efficient alignment of short DNA sequences to the human genome. Genome Biol. 2009;10(3):R25

~~36.~~43. Kent WJ. BLAT–the BLAST-like alignment tool. Genome Res. 2002;12(4):656–64.

~~37.~~44. Simao FA, Waterhouse RM, Ioannidis P, Kriventseva EV, Zdobnov EM. BUSCO: assessing genome assembly and annotation completeness with single-copy orthologs. Bioinformatics. 2015;31(19):3210–2.

~~38.~~45. Supporting data for "Long-read sequencing and *de novo* genome assembly of *Ammopiptanthus nanus*, a desert shrub ". GigaScience Database. 2017. http://dx.doi.org/**.****/******

Tables

~~**Table 1. PacBio subreads used for the *A. nanus* genome assembly.**~~

~~Type~~	~~Read bases (bp)~~	~~Reads number~~	~~Read N50 (bp)~~	~~Mean length (bp)~~
~~Subread~~	~~64,538,018,400~~	~~7,918,322~~	~~12,786~~	~~8,150~~

~~**Table 2. Statistics of the *A. nanus* assembly**~~

~~Contig number~~	~~Contig length (bp)~~	~~Contig N50 (bp)~~	~~Contig N90 (bp)~~	~~Contig max (bp)~~	~~GC content (%)~~	~~Gap total length (bp)~~
~~1,099~~	~~823,653,484~~	~~2,761,160~~	~~562,763~~	~~11,314,623~~	~~36.74~~	~~0~~

Table 1~~3~~ Summary of *A. nanus* genome annotation

Method	Software and gene set	Gene number
Ab initio based	Genscan	26,686
	Augustus	32,931
	GlimmerHMM	43,252
	GeneID	45,622
	SNAP	58,443
Homology based	GeMoMa	
	Arachis duranensis	98,866
	Cicer arietinum	54,840
	Phaseolus vulgaris	56,919
	Glycine max	97,568
RNA-seq based	PASA	43,789
	TransDecoder	43,215
	GeneMarkS-T	30,748
Integration	EVM	37,259

~~**Table 4 Statistics of the genome annotation**~~

~~Gene number~~	~~Gene Length (bp)~~	~~Avery gene length (bp)~~	~~CDS length (bp)~~	~~Avery CDS length (bp)~~	~~Intron length (bp)~~	~~Avery intron length (bp)~~
~~37,259~~	~~173,484,514~~	~~4,656.18~~	~~42,040,206~~	~~1,128.32~~	~~118,059,238~~	~~3168.61~~

Table ~~5~~2 Summary of functional annotation for the predicted genes

Annotation database	Annotated gene number	Percentage (%)
GO	20,236	54.31
KEGG	10,169	27.29
KOG	18,294	49.09
Pfam	26,793	71.91
Swissprot	21,461	57.59
TrEMBL	35,021	93.99
NR	34,984	93.89
Nt	34,124	91.58
All Annotated	36,033	96.70

~~**Table 6 Statistics of the predicted pseudogenes**~~

~~Pseudogene number~~	~~Total length (bp)~~	~~Average length (bp)~~
~~7,588~~	~~20,322,021~~	~~2,678.17~~

~~**Table 7 The alignment of the Illunima reads to the *A. nanus* genome assembly**~~

~~Library~~	~~Total reads~~	~~Mapped (%)~~	~~Concordantly mapped (%)~~
~~350 bp~~	~~373,513,096~~	~~100~~	~~98.07~~

Table ~~8~~3 The alignment of the unigenes to the *A. nanus* genome assembly

Range of length	Total number	Aligned number	Percentage (%)
≥~~>=~~500	81,429	81,429	100
≥~~>=~~1,000	54,385	54,385	100

~~**Table 9 BUSCO assessment of the *A. nanus* genome assembly**~~

~~Complete BUSCOs~~	~~Complete and single-copy BUSCOs~~	~~Complete and duplicated BUSCOs~~	~~Fragmented BUSCOs~~	~~Missing BUSCOs~~
~~1,328~~	~~1,239~~	~~89~~	~~35~~	~~77~~

Figure legends

Figure 1. A flowering *A. nanus*

~~Figure 2. Venn diagram showing the overlap of the gene annotation results of the three categories of gene prediction methods~~

Figure 1

Click here to download Figure Figure_1.jpg

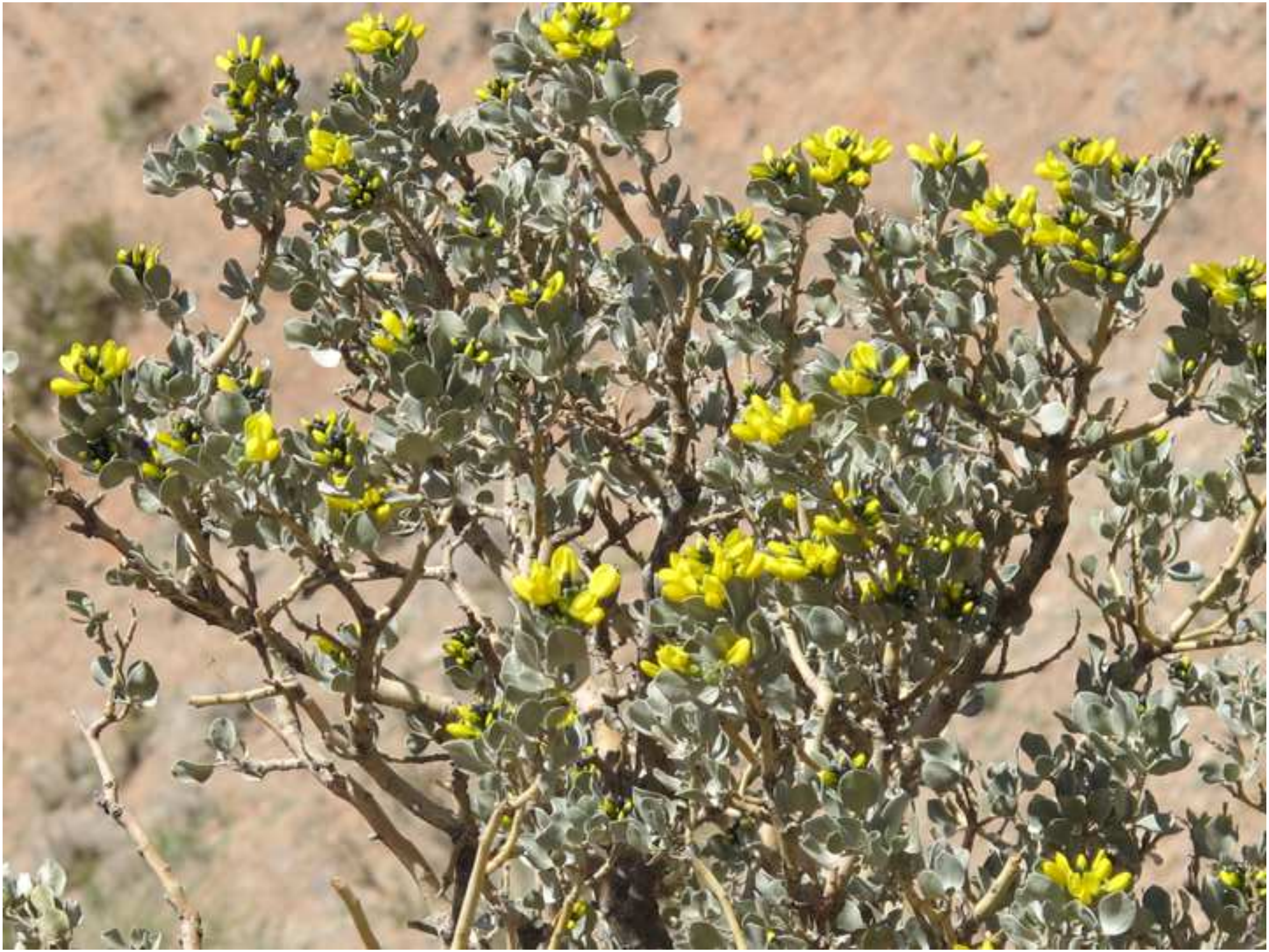

Click here to access/download
Supplementary Material
Supplementary material_20171219_1.docx
