# Supplementary material for: Long-read sequencing and de novo genome assembly of Ammopiptanthus nanus, a desert shrub
Source: Gigascience. 2018 Jun 28;7(7):giy074. doi: 10.1093/gigascience/giy074 (PMC6048559; doi:10.1093/gigascience/giy074)
Supplement: GIGA-D-17-00264_Revision_2.pdf [file giy074_giga-d-17-00264_revision_2.pdf]

GigaScience

Long-read sequencing and de novo genome assembly of Ammopiptanthus nanus, a desert shrub

--Manuscript Draft--

Manuscript Number:	GIGA-D-17-00264R2
Full Title:	Long-read sequencing and de novo genome assembly of Ammopiptanthus nanus, a desert shrub
Article Type:	Data Note
Funding Information:	the National Natural Science Foundation of China (31770363) — Prof. Yijun Zhou<br>the National Natural Science Foundation of China (31670335) — Dr. Fei Gao<br>the Ministry of Education of China through 111 projects (B08044) — Prof. Yijun Zhou<br>the Ministry of Education of China through 985 projects (YLDX01013) — Prof. Yijun Zhou
Abstract:	Background<br>Ammopiptanthus nanus is a rare broad-leaved shrub in the desert and arid regions of Central Asia. This plant species exhibits extremely high tolerance to drought and freezing and has been used in abiotic tolerance research in plants. As a relic of the Tertiary period, A. nanus is of great significance to plant biogeographic research in the ancient Mediterranean region. Here we report a draft genome assembly using the PacBio platform and gene annotation for A. nanus.<br>Findings<br>A total of 64.72 gigabases (Gb) of raw PacBio Sequel reads were generated from four 20 kb libraries. After filtering, 64.53 Gb of clean reads were obtained, giving 72.59 × coverage depth. Assembly using Canu gave an assembly length of 823.74 Mb, with a contig N50 of 2.76 Mb. The final size of the assembled A. nanus genome was close to the 889 Mb estimated by k-mer analysis. The gene annotation completeness was evaluated by BUSCO, and 1,327 out of the 1,440 conserved genes (92.15%) could be found in the A. nanus assembly. Genome annotation revealed that 74.08% of the A. nanus genome is composed of repetitive elements, and 53.44% of long terminal repeat elements (LTRs). We predicted 37,188 protein-coding genes, of which 96.53% were functionally annotated.<br>Conclusions<br>The genomic sequences of A. nanus could be a valuable source for comparative genomic analysis in the legume family, and will be useful for understanding the phylogenetic relationships of the Thermopsideae and the evolutionary response of plant species to the Qinghai Tibetan Plateau uplift.
Corresponding Author:	Fei Gao<br>Minzu University of China<br>Beijing, CHINA
Corresponding Author Secondary Information:	
Corresponding Author's Institution:	Minzu University of China
Corresponding Author's Secondary Institution:	
First Author:	Fei Gao
First Author Secondary Information:	
Order of Authors:	Fei Gao
	Xue Wang

Powered by Editorial Manager® and ProduXion Manager® from Aries Systems Corporation

	Xuming Li
	Mingyue Xu
	Huayun Li
	Merhaba Abla
	Huigai Sun
	Shanjun Wei
	Jinchao Feng
	Yijun Zhou
**Order of Authors Secondary Information:**	
**Response to Reviewers:**	Author's Response to Reviewer Comments

Dear editors and reviewers,

Enclosed you will find the manuscript GIGA-D-17-00264-R2, the revised version of the originally submitted paper entitled "Long-read sequencing and de novo genome assembly of Ammopiptanthus nanus, a desert shrub" that we are re-submitting for publication in GigaScience as Data Note.

Thank you for the opportunity to revise the manuscript. We have re-run the assembly polishing by adding a polishing step with Arrow prior to Pilon polishing. All the subsequent analysis, including the repeat annotation and gene prediction, and assessment of the genome assembly, were also conducted again.

We have responded to all comments and suggestions made by the reviewers. Our responses to each comment are given below and changes in the revised manuscript are shown using track changes. We sincerely appreciate the reviewers' comments that really helped us improving the genome assembly and the manuscript and we hope that all the issues raised were addressed at satisfaction.

Thank you for your consideration. We look forward to hearing from you soon.

Regards
Fei Gao (on behalf of all co-authors)
College of life and environmental sciences,
Minzu University of China, Beijing, 100081, China
E-mail: gaofei@muc.edu.cn.

Reviewer #2: The authors have clarified important issues and questions raised by the reviewers and added helpful citations.

Minor points:
1. Even though the authors stated that they have deposited the data as Gigascience requested, an additional genome browser will be still very helpful for users to navigate this genome as a reference. I would suggest the authors to work with other plant genomics databases (PlantDB, Gramene and so on) to make their genome more accessible to users.

Reply: Thank you for your advice. We will contact the staff of PlantGDB or other genomics databases to further release our genome data after acceptance of this article.

2. Line 27-28: BUSCO can only estimate the completeness of gene space. The statement of "the genome completeness" is inaccurate. Please revise.

Reply: The statement of "the genome completeness was evaluated by BUSCO " was replaced by "the gene annotation completeness was evaluated by BUSCO"

Powered by Editorial Manager® and ProduXion Manager® from Aries Systems Corporation

3. Line 65: N50 is only a statistic to measure assemblies. Please revise this sentence to "these assemblies generally content very fragmented sequences", or something similar.

Reply: The statement of " these assemblies generally have low N50 values and a large number of contigs" was replaced by “these assemblies generally contain very fragmented sequences”

Reviewer #3: The revised manuscript addressed most of my comments properly. Two major and one minor concerns however remain open.

2) The genome was assembled with Canu and polished with Pilon. Could the authors explain while the initial Canu assembly was not polished with Arrow prior to Pilon polishing. A lot of medium sized InDels usually remain in the assembly if only Pilon is used for assembly polishing. The final genome release should include a Arrow polishing step.

The authors response to my comment and a similar one from reviewer 2 (5th comment) is not satisfying. Yes, long read polishing is not increasing assembly quality to the same extent as a final short read polishing. But long read polishing removes spurious insertions and deletions at a length were short reads fail. The authors obviously did not understand that each polishing method addresses different types of assembly errors and therefore both methods should be applied in modern genomes projects, beginning with long reads and ending with short reads. The manuscript should specifically state that a long read polishing was not applied, and longer spurious insertion and deletions introduced during assembly might not be corrected.

Reply: Thank you for your constructive advice. We have re-run the assembly polishing by adding a polishing step with Arrow prior to Pilon polishing. All the subsequent analysis, including the repeat annotation and gene prediction, and assessment of the genome assembly, were also conducted again. We have updated the related dataset and the statistical tables.

6) Please indicate whether default mapping parameters were used to assess the genome completeness via short read mappings and adopt the quality assessment method from (Bickhart et al. 2017, Nat Genet.; Jain et al. 2017, bioRxiv).

The second concern is the error evaluation analysis the authors applied. They did not what I asked for and/or misunderstood the Bickhart et al. 2017, Nat Genet. reference I added. Authors simply reported re-mapping ratios instead of calling SNPs with the re-mappings and inferring Q-value as pointed out by Bickhart et al. The manuscript should contain a sentence that only completeness was tested but accuracy of the assembly was not specifically assessed or, and that would be my preferred option, the authors should re-run the analysis. A simple workflow can be taken from https://github.com/fbemm/onefc-oneasm/wiki/Assembly-Validation.

Reply: The SNP-based assembly quality assessment was performed referring the method provided by Bickhart et al and the erroneous bases in the genome assembly were identified using the variant calling software FreeBayes with default parameters. The QV value of our genome assembly was calculated out to be 38.95, which shows that the genome quality in base level is good.

3) The size of the genome was estimated via k-mer distribution. Figure S1 shows a major and a minor peak. I agree with the authors that the highest peak represents the diploid genome given the low amounts of BUSCO duplicates. Nevertheless, a in-depth description of the repetitve peak (better contigs associated with it) would be interesting and should be added to the manuscript. Please describe how much contigs and how much total sequence are duplicated (showing k-mers from the second peak) and elaborate on the annotated genes/repeats you can find of there. Consider moving the k-mer histogram and maybe a graphical (GO enrichment based) summary of duplicated genes/repeats to the main text as figure.

Authors misunderstood my request. The idea was, to isolates k-Mers from the k-Mer

Powered by Editorial Manager® and ProduXion Manager® from Aries Systems Corporation

	distribution (can be simply done with Jellyfish dump by specifying a low and high cutoff that fits to the 2nd peak) and use them as baits to isolate contigs that contain these k-Mer (can be done using "bbduk2 in=contigs.fasta out=baited.fasta ref=2nd-peak-kmers-fasta hdist=0 mm=f). It would have been interesting to see if these elements are a) properly assembled, thus appear on contigs with regions from the first peak or b) are simply collapsed during the assembly. On top of that an repeat/annotation analysis could have revealed the nature of the contigs and thereby have helped to understand any functional implication of this partial genome duplication. It is up to the authors to add that analysis, but it would have improved the understand of the A. nanus genome for sure.<br><br>Reply: Thank you for your advice, but we met some difficulties in conducting the analysis you recommend and failed to complete the analysis.
**Additional Information:**	
**Question**	**Response**
Are you submitting this manuscript to a special series or article collection?	No
**Experimental design and statistics**<br><br>Full details of the experimental design and statistical methods used should be given in the Methods section, as detailed in our Minimum Standards Reporting Checklist. Information essential to interpreting the data presented should be made available in the figure legends.<br><br>Have you included all the information requested in your manuscript?	Yes
**Resources**<br><br>A description of all resources used, including antibodies, cell lines, animals and software tools, with enough information to allow them to be uniquely identified, should be included in the Methods section. Authors are strongly encouraged to cite Research Resource Identifiers (RRIDs) for antibodies, model organisms and tools, where possible.<br><br>Have you included the information requested as detailed in our Minimum Standards Reporting Checklist?	Yes
**Availability of data and materials**<br><br>All datasets and code on which the conclusions of the paper rely must be either included in your submission or deposited in publicly available repositories (where available and ethically appropriate), referencing such data using	Yes

a unique identifier in the references and in the “Availability of Data and Materials” section of your manuscript.<br><br>Have you have met the above requirement as detailed in our Minimum Standards Reporting Checklist?	

Powered by Editorial Manager® and ProduXion Manager® from Aries Systems Corporation

Manuscript
Click here to access/download;Manuscript;DesertShrub_10-6-18.docx
Click here to view linked References

Long-read sequencing and *de novo* genome assembly of *Ammopiptanthus nanus*, a desert shrub

Fei Gao[1], Xue Wang[1], Xuming Li[2], Mingyue Xu[2], Huayun Li[3], Merhaba Abla[1], Huigai Sun[1], Shanjun Wei[1], Jinchao Feng[1], Yijun Zhou[1*]

[1]College of Life and Environmental Sciences, Minzu University of China, Beijing, 100081, China

[2]Biomarker Technologies Corporation, Beijing, 101300, China.

[3]Annoroad Genomics, Beijing, 100176, China

Email addresses: Fei Gao <gaofei@muc.edu.ecn>, Xue Wang <wangxue@muc.edu.cn>, Xuming Li<lixm@biomarker.com.cn>, Mingyue Xu<xumy@biomarker.com.cn>, Huayun Li < huayunli@annoroad.com >, Abla Merhaba< Merhaba@muc.edu.cn >, Huigai Sun <sunhuigai66@163.com>, Shanjun Wei<wei.s.j@163.com>, Jincho Feng<fengjinchao@muc.edu.cn>

*Correspondence should be addressed to: Y. Z. <zhouyijun@muc.edu.cn>

Abstract

Background

Ammopiptanthus nanus is a rare broad-leaved shrub in the desert and arid regions of Central Asia. This plant species exhibits extremely high tolerance to drought and freezing and has been used in abiotic tolerance research in plants. As a relic of the Tertiary period, *A. nanus* is of great significance to plant biogeographic research in the ancient Mediterranean region. Here we report a draft genome assembly using the PacBio platform and gene annotation for *A. nanus*.

Findings

A total of 64.72 gigabases (Gb) of raw PacBio Sequel reads were generated from four 20 kb libraries. After filtering, 64.53 Gb of clean reads were obtained, giving 72.59 × coverage depth. Assembly using Canu gave an assembly length of 823.74 Mb, with a contig N50 of 2.76 Mb. The final size of the assembled *A. nanus* genome was close to the 889 Mb estimated by k-mer analysis. The gene annotation completeness was evaluated by BUSCO, and 1,327 out of the 1,440 conserved genes (92.15%) could be found in the *A. nanus* assembly. Genome annotation revealed that 74.08% of the *A. nanus* genome is composed of

repetitive elements, and 53.44% of long terminal repeat elements (LTRs). We predicted 37,188 protein-coding genes, of which 96.53% were functionally annotated.

Conclusions

The genomic sequences of *A. nanus* could be a valuable source for comparative genomic analysis in the legume family, and will be useful for understanding the phylogenetic relationships of the Thermopsideae and the evolutionary response of plant species to the Qinghai Tibetan Plateau uplift.

Keywords

Ammopiptanthus nanus, PacBio sequencing, Genome assembly, Genome annotation

Data Description

Background information

Ammopiptanthus nanus, a desert shrub and a relic from the tertiary period, is one of two species in the genus *Ammopiptanthus*. This genus belongs to the tribe Thermopsideae and the family Fabaceae (Figure 1). *Ammopiptanthus* is the only genus of evergreen broadleaf shrub distributed in the desert and arid regions of Central Asia, and the plants in this genus play important ecological roles by fixing moving sands and delaying further desertification [1].

Tribe Thermopsideae is considered to be a basal branch in the family Fabaceae and the habitats of the ca. 45 plant species in tribe Thermopsideae are interspersed among the Mediterranean Basin, Central Asia, and temperate North America. Studies on the molecular biology of these plant species will promote understanding of the phylogeny of family Fabaceae, as well as some interesting biogeographical topics, such as how the Qinghai-Tibetan Plateau uplift and Tethys retreat affected plant evolution [2, 3]. In addition, the genus *Ammopiptanthus* is a unique and isolated branch in tribe Thermopsideae. There are still some debates about the evolution and phylogeny of this genus [3], and more molecular evidence is needed to clarify these issues.

Species in genus *Ammopiptanthus* exhibit extremely high tolerance to drought and freezing and have been used in abiotic tolerance research in plants [4–6]. Although several transcriptome analyses of the response to drought and cold stress have been conducted [1, 7–9], the lack of genome sequence information impedes further investigation into the molecular mechanism underlying the stress tolerance of *Ammopiptanthus* species.

Most of the *de novo* assemblies of plant genomes recently reported have been performed using the next generation sequencing technologies such as Illumina or 454 sequencing platforms [10–12]. However, these assemblies generally contain very fragmented sequences, partly because of the complexity of the plant genome. The newly developed Pacific BioSciences (PacBio) sequencing platform, a third-generation sequencing technology, has started to address some of the intrinsic challenges in sequencing and assembling large and complex plant genomes, via producing tens of thousands of long individual reads (up to ~40 kb) [13]. Recently, several complicated plant genomes, including those of maize [14], sunflower [15], and *Chenopodium quinoa* [16], have been sequenced using the PacBio sequencing technology. In the present study, we employed single molecule real-time (SMRT) sequencing developed by PacBio, to generate a draft genome assembly for *A. nanus*.

Sample collection and genomic DNA sequencing

The leaf tissues of a single *A. nanus* tree (NCBI Taxonomy ID: 111851) were collected from Xinjiang, China. After collection, tissues were immediately transferred into liquid nitrogen and stored until DNA extraction. The extraction of DNA was conducted using the CTAB method according to the protocol ‘Preparing *Arabidopsis* Genomic DNA for Size-Selected ~20 kb SMRTbell™ Libraries’ (http://www.pacb.com/wp-content/uploads/2015/09/Shared-Protocol-Preparing-Arabidopsis-DNA-for-20-kb-SMRTbell-Libraries.pdf). The quality of the extracted genomic DNA was checked by 1% agarose gel electrophoresis, and the concentration was quantified using a Qubit fluorimeter (Invitrogen, Carlsbad, CA, USA).

Long-read sequencing was performed at Biomarker Technologies Corporation (Beijing, China) with a PacBio Sequel sequencer (Pacific Biosciences, Menlo Park, CA, USA). The SMRT Bell library was prepared using a DNA Template Prep Kit 1.0 (PacBio p/n 100-259-100) and four 20 kb SMRTbell libraries were constructed. Genomic DNA (10 μg) was mechanically sheared using a Covaris g-Tube (Kbiosciences p/n 520079) with a goal of DNA fragments of approximately 20 kb. A Bioanalyzer 2100 12K DNA Chip assay (Agilent p/n 5067-1508) was used to assess the fragment size distribution. Sheared genomic DNA (5 μg) was DNA-damage repaired and end-repaired using polishing enzymes. A blunt-end ligation reaction followed by exonuclease treatment was conducted to generate the SMRT Bell template. A Blue Pippin device (Sage Science, Inc., Beverly, MA, USA) was used to size select the SMRT Bell template and enrich large fragments (> 10 kb). The size-selected library was quality inspected and quantified on an Agilent Bioanalyzer 12 kb DNA Chip (Agilent Technologies, Santa Clara, CA, USA) and a Qubit fluorimeter (Invitrogen, Carlsbad, CA, USA). A ready-to-sequence SMRT Bell-Polymerase Complex was created using a Binding Kit 2.0 (PacBio p/n 100-862-200), according to the manufacturer's instructions. The Sequel instrument was programmed to load and sequence the sample on PacBio SMRT cells v3.0 (PacBio p/n 100-171-800), acquiring one movie of 360 min per SMRT cell. The MagBead loading (PacBio p/n 100-125-900) method was employed to improve the enrichment of the larger fragments. A total of 13 SMRT cells were processed yielding 64.72 G subread sequences.

For Illumina sequencing, paired-end libraries with insert sizes of 350 bp were constructed with the standard protocol provided by Illumina (San Diego, CA, USA) and sequenced on an Illumina HiSeq X ten platform. A total of 55.97 Gb of paired-end (2 × 150 bp) clean sequences were generated (Table S1). These data were used for genome size estimation, correction of genome assembly, and assembly evaluation.

Genome size estimation

We characterized the genome size and heterozygosity using the distribution of k-mers of length 19 from the Illumina HiSeq reads (55.97 Gb clean reads from 350 bp insert size library, NCBI SRA accession number: SRX3286209). This analysis was performed using "kmer_freq_stat" software (developed by Biomarker Technologies). The genome size (G) of *A. nanus* was estimated by the following formula: G = k-mer

number/average k-mer depth, where k-mer number = total k-mers - abnormal k-mers (with too low or too high frequency). The highest peak in the k-mer distribution curve was found at the k-mer depth of 53, with a k-mer number of 47,408,863,457 (Figure S1). The peak at depth of more than 106 was a repetitive peak (k-mers duplicated because of repetition). Finally, the *A. nanus* genome size was estimated to be 888.92 Mb, the heterozygosity was approximately 0.02%, and the data used in 19-mer analysis was approximately 53× coverage of the genome.

Genome assembly

The Sequel raw bam files were converted into subreads in fasta format using the standard PacBio SMRT software package (read data is available at the NCBI SRA accession number: SRX3262947). Then subreads of less than 500 bp were filtered out. Finally, 7,918,322 reads and 64,538,018,400 bases (~ 73 × depth) were produced. The average subread length was 8.15 kb with a N50 length of 12.79 kb (Table S2). The genome assembly was conducted using Canu software (v1.5) [17] (correctedErrorRate=0.045, corOutCoverage=70). The draft genome was polished with Arrow (SMRT link v5.0.1, --minCoverage 15) using all SMRT reads and polished by Pilon v1.22 (Pilon, RRID:SCR_014731) [18] using the Illumina reads with the default settings . Finally, we assembled a genome of 823.74 Mb with 1,099 contigs and contig N50 of 2.76 Mb (Table S3).

Repeat annotation and gene prediction

For repeat detection, first, four software packages, i.e., LTR-FINDER (v1.0.5) [19], MITE-Hunter (v1.0.0) [20], PILER (v1.0) [21], and RepeatScout v1.0.5, (RepeatScout ,RRID:SCR_014653) [22] were used to build a *de novo* repeat library on the basis of our assembly with the default settings, and then, the predicted repeats were classified using PASTEClassifier (v1.0) [23] and merged with Repbase (19.06) [24]. Finally, using the resulting repeat database as the final repeat library, RepeatMasker v4.0.5 (RepeatMasker, RRID:SCR 012954) [25] was utilized to identify repetitive sequences in the *A. nanus* genome with the following parameters “-nolow -no_is -norna -engine wublast”. Overall, approximately 610.25 Mb of repetitive sequences (74.08% of the assembly) were detected, containing 440.18 Mb (53.44% of the assembly) LTRs (Table S4).

Ab initio-based, homolog-based, and RNA-seq-based gene prediction methods were conducted in combination to identify the protein-coding genes in the *A. nanus* genome assembly. Genscan [26], Augustus v2.4, (Augustus, RRID:SCR_008417) [27], GlimmerHMM v3.0.4, (GlimmerHMM, RRID:SCR_002654) [16], GeneID (v1.4) [28], and SNAP v2006-07-28 (SNAP, RRID:SCR_002127) [29] with the default parameters were employed for the *Ab initio* based gene prediction, and all these software packages were trained using the *Arabidopsis* gene model before gene prediction. For gene prediction using Augustus, besides the Arabidopsis's gene model, the PASA's gene model was also used as initial gene model for training. Finally, the best gene model with higher accuracy and specificity was used. Quality evaluation of gene models was conducted by aligning transcriptome sequences to the whole genome assembly using Tophat (Table S5). GeMoMa (v1.3.1) [30] was used in homolog-based gene annotation and the protein database of *Cicer arietinum* (GCA_000331145.1), *Phaseolus vulgaris* (GCA_000499845.1), *Glycine max* (GCA_000004515.3), and *Arachis duranensis* (GCA_000817695.2) from GenBank were used as the reference databases. For the RNA-seq-based method of gene prediction, TransDecoder (v2.0, http://transdecoder.github.io), GeneMarkS-T v5.1 (RRID:SCR_011930) [31], and PASA v2.0.2, (RRID:SCR_014656) [32] were used, and the *A. nanus* transcriptome data were assembled in a previous study (NCBI SRA accession number: SRX1409432 and SRX1406652) [33]. Finally, the results from the three methods were integrated using EVM (v1.1.1, RRID:SCR_014659) [34]. Higher weights were assigned to the PASA predicted transcripts from unigenes and GeMoMa predicted homologous transcripts than to the *ab initio* predicted transcripts when conducting the EVM integration. In total, a gene set with 37,144 protein-coding genes was predicted from the *A. nanus* genome assembly (Table 1 and S6, and Figure S2). These genes were scattered over 1,099 contigs, averaging 33.80 genes per contig. The genes were annotated by aligning to the NR, Nt, KOG [35], KEGG (KEGG, RRID:SCR_001120) [30], Swissprot (Swissprot, RRID:SCR_002380) [36], TrEMBL [37] databases using blast with an e-value cutoff of 1E-5 and also aligned to the Pfam (Pfam, RRID:SCR_004726) database [38] using hmmer V3.0 (-E 0.00001 --domE 0.00001 --cpu 2 --noali --acc) [39]. GO terms were assigned to the genes using Blast2GO pipeline [40]. In all, 96.71% of the predicted genes could be classified into families according to their putative functions (Table 2).

For pseudogene prediction, first, GenBlastA [41] was used to scan the *A. nanus* genome for sequences homologous to the known protein-coding genes it contained, then GeneWise (GeneWise, RRID:SCR_015054) [42] was adopted to search the premature stop codons or frameshift mutations in those sequences and, consequently, to identify pseudogenes. In total, 7,891 pseudogenes were identified from the *A. nanus* genome (Table S7).

Assessment of the genome assembly

First, the 55.97G Illumina sequencing reads (NCBI SRA accession number: SRX3286209) used for k-mer analysis were aligned to the *A. nanus* genome assembly using bowtie [43]. The results showed that all Illumina reads were mapped and 98.45% PE reads were mapped concordantly (Table S8). Using these short reads, the estimated quality value (QV) of *A. nanus* genome was calculated according to a previously described method [44, 45], and the erroneous bases in the genome assembly were identified using the variant calling software FreeBayes v0.9.14 (FreeBayes, RID:SCR_010761) with default parameters. The QV of *A. nanus* genome was estimated to be 38.95, which means that the accuracy of the assembly in base level is fine after base correction.

Second, the *A. nanus* unigenes assembled in a previous study (NCBI SRA accession number: SRX1409432 and SRX1406652) [33] were aligned to the *A. nanus* genome using BLAT v0.36 (BLAT, RRID:SCR_011919) [46] with default parameters. The alignment indicated that 100% of unigenes (≥ 500 bp in length) assemblies were mapped to the *A. nanus* genome assembly (Table 3).

We also evaluated the completeness of the genome assembly of *A. nanus* by using BUSCO v2.0 (BUSCO, RRID:SCR_015008) [47]. The results showed that 9215% (1,327 out of 1,440 BUSCOs) of plant sets (embryophyta_odb9, download from http://busco.ezlab.org/) were identified as complete in the *A. nanus* assembly (Table S9). Together, the results indicated that our dataset represented a genome assembly with a high level of coverage.

Conclusion

In summary, the draft genome sequence of *A. nanus* obtained in the present study demonstrated that third-generation sequencing technology, such as the PacBio platform, could be useful in deciphering complex plant genomes. The availability of the *A. nanus* genome sequence should facilitate *de novo* genome assembly of other species in this genus. The datasets from the present manuscript could not only provide a valuable source for further comparative genomics analysis in the legume family and help to answer some important questions related to the biogeography in the ancient Mediterranean region, but also facilitate understanding of how plants adapt to the stressful conditions in temperate deserts in Central Asia.

List of abbreviations

Gb: Giga base; TE: Transposable element; GO: Gene Ontology; PE: paired-end; LTR: Long terminal repeat element; CDS: Coding DNA sequence; SMRT: Single molecule real-time; PacBio: Pacific BioSciences; KOG: Eukaryotic Orthologous Groups of proteins; KEGG: Kyoto Encyclopedia of Genes and Genomes

Competing interests

The authors declare that they have no competing interests.

Funding

This work was financially supported by the National Natural Science Foundation of China (31670335 and 31770363) and the Ministry of Education of China through 111 and 985 projects (B08044, YLDX01013).

Availability of Supporting Information

Raw genomic sequence reads are available in the NCBI Sequence Read Archive under project number PRJNA413722. Supporting data are available from the *GigaScience* database GigaDB [48].

Author Contributions

YZ and JF oversaw the project. MA, XW, and SW collected the sample and extracted the genomic DNA. XL, HL, MX, and HS performed the genome assembly, annotated the genome and analyzed data. FG analyzed data. FG and YZ wrote the manuscript.

References

1. Gao F, Wang J, Wei S, Li Z, Wang N, Li H. Transcriptomic analysis of drought stress responses in *Ammopiptanthus mongolicus* leaves using the RNA-Seq technique. Plos ONE. 2015;10(4):e0124382.
2. Zhang ML, Huang JF, Sanderson SC, Yan P, Wu YH, Pan BR. Molecular biogeography of tribe Thermopsideae (Leguminosae): A Madrean-Tethyan disjunction pattern with an African origin of core Genistoides. Biomed Res Int. 2015; 2015:864804.
3. Shi W, Liu PL, Duan L, Pan BR, Su ZH. Evolutionary response to the Qinghai-Tibetan Plateau uplift: phylogeny and biogeography of *Ammopiptanthus* and tribe Thermopsideae (Fabaceae). Peer J. 2017;5:e3607.
4. Xu S, An L, Feng H, Wang X, Li X. The seasonal effects of water stress on *Ammopiptanthus mongolicus* in a desert environment. J Arid Environ. 2002; 51(3):437–47.
5. Wang W, Chen Y, Liu M, Lu C. Effects of cold-hardening on compatible solutes and antioxidant enzyme activities related to freezing tolerance in *Ammopiptanthus mongolicus* seedlings. For Stud China. 2008;10(2):101–6.
6. Gao T-P, Chen T, Feng H-Y, An L-Z, Xu S-J, Wang X-L. Seasonal and annual variation of osmotic solute and stable carbon isotope composition in leaves of endangered desert evergreen shrub *Ammopiptanthus mongolicus*. S Afr J Bot. 2006; 72(4):570–8.
7. Zhou Y, Gao F, Liu R, Feng J, Li H. *De novo* sequencing and analysis of root transcriptome using 454 pyrosequencing to discover putative genes associated with drought tolerance in *Ammopiptanthus mongolicus*. BMC Genomics. 2012;13:266.
8. Wu Y, Wei W, Pang X, Wang X, Zhang H, Dong B, et al. Comparative transcriptome profiling of a desert evergreen shrub, *Ammopiptanthus mongolicus*, in response to drought and cold stresses. BMC Genomics. 2014;15:671.

9. Pang T, Ye CY, Xia X, Yin W. *De novo* sequencing and transcriptome analysis of the desert shrub, *Ammopiptanthus mongolicus*, during cold acclimation using Illumina/Solexa. BMC Genomics. 2013;14:488.

10. Fu Y, Li L, Hao S, Guan R, Fan G, Shi C, et al. Draft genome sequence of the Tibetan medicinal herb Rhodiola crenulata. Gigascience. 2017;6(6):1–5.

11. Zhao D, Hamilton JP, Pham GM, Crisovan E, Wiegert-Rininger K, Vaillancourt B, et al. *De novo* genome assembly of *Camptotheca acuminata*, a natural source of the anti-cancer compound camptothecin. Gigascience. 2017;6(9):1–7.

12. Xia EH, Zhang HB, Sheng J, Li K, Zhang QJ, Kim C, et al. The tea tree genome provides insights into tea flavor and independent evolution of caffeine biosynthesis. Mol Plant. 2017;10(6):866–77.

13. Roberts RJ, Carneiro MO, Schatz MC. The advantages of SMRT sequencing. Genome Biol. 2013;14(7):405.

14. Jiao Y, Peluso P, Shi J, Liang T, Stitzer MC, Wang B, et al. Improved maize reference genome with single-molecule technologies. Nature. 2017;546(7659):524–7.

15. Badouin H, Gouzy J, Grassa CJ, Murat F, Staton SE, Cottret L, et al. The sunflower genome provides insights into oil metabolism, flowering and Asterid evolution. Nature. 2017;546(7656):148–52.

16. Jarvis DE, Ho YS, Lightfoot DJ, Schmöckel SM, Li B, Borm TJ, et al. The genome of *Chenopodium quinoa*. Nature. 2017;542(7641):307–12.

17. Koren S, Walenz BP, Berlin K, Miller JR, Bergman NH, Phillippy AM. Canu: scalable and accurate long-read assembly via adaptive k-mer weighting and repeat separation. Genome Res. 2017;27(5):722–36.

18. Walker BJ, Abeel T, Shea T, Priest M, Abouelliel A, Sakthikumar S, et al. Pilon: an integrated tool for comprehensive microbial variant detection and genome assembly improvement. PLoS ONE. 2014;9(11):e112963.

19. Xu Z, Wang H. LTR˙FINDER: an efficient tool for the prediction of full-length LTR retrotransposons. Nucleic Acids Res 2007;35(web server issue):W265–8.

20. Han Y, Wessler SR. MITE-Hunter: a program for discovering miniature inverted-repeat transposable elements from genomic sequences. Nucleic Acids Res. 2010;38(22):e199.

21. Edgar RC, Myers EW. PILER: identification and classification of genomic repeats. Bioinformatics. 2005;21:i152–8.

22. Price AL, Jones NC, Pevzner PA. *De novo* identification of repeat families in large genomes. Bioinformatics 2005;21 (suppl 1):i351–8.

23. Hoede C, Arnoux S, Moisset M, Chaumier T, Inizan O, Jamilloux V, et al. PASTEC: An automatic transposable element classification tool. PLoS ONE. 2014;9:e91929.

24. Bao W, Kojima KK, Kohany O. Repbase Update, a database of repetitive elements in eukaryotic genomes. Mobile DNA. 2015;6:11.

25. Tarailo-Graovac M, Chen N. Using RepeatMasker to identify repetitive elements in genomic sequences. Curr Protoc Bioinformatics. 2009; Chapter 4: Unit 4.10.

26. Burge C, Karlin S. Prediction of complete gene structures in human genomic DNA. J Mol Biol. 1997; 268:78–94.

27. Stanke M, Waack S. Gene prediction with a hidden Markov model and a new intron submodel. Bioinformatics. 2003;19 Suppl 2:ii215–25.

28. Blanco E, Parra G, Guigó R: Using geneid to identify genes. Curr Protoc Bioinformatics. 2007;4.3.

29. Korf I. Gene finding in novel genomes. BMC bioinformatics. 2004;5:59.

30. Keilwagen J, Wenk M, Erickson JL, Schattat, MH, Jan, G, Frank, H. Using intron position conservation for homology-based gene prediction. Nucleic Acids Res. 2016;44:e89.

31. Tang S, Lomsadze A, Borodovsky M. Identification of protein coding regions in RNA transcripts. Nucleic Acids Res. 2015;43(12):e78.

32. Campbell MA, Haas BJ, Hamilton JP, Mount SM, Buell CR. Comprehensive analysis of alternative splicing in rice and comparative analyses with *Arabidopsis*. BMC genomics. 2006;7:327.

33. Gao F, Li H, Xiao Z, Wei C, Feng J, Zhou Y, *De novo* transcriptome analysis of *Ammopiptanthus nanus* and its comparative analysis with *A. mongolicus*. Trees. 2018;32(1):287–300.

34. Haas BJ, Salzberg SL, Zhu W, Pertea M, Allen JE, Orvis J, et al. Automated eukaryotic gene structure annotation using EVidenceModeler and the Program to Assemble Spliced Alignments. Genome Biol. 2008;9(1):R7.

35. Tatusov RL, Natale DA, Garkavtsev IV, Tatusova TA, Shankavaram UT, Rao BS, et al. The COG database: new developments in phylogenetic classification of proteins from complete genomes. Nucleic Acids Res. 2001;29(1):22–8.

36. Kanehisa M, Goto S. KEGG: Kyoto Encyclopedia of Genes and Genomes. Nucleic Acids Res. 2000;28(1):27–30.

37. Boeckmann B, Bairoch A, Apweiler R, Blatter M-C, Estreicher A, Gasteiger E, et al. The SWISS-PROT protein knowledgebase and its supplement TrEMBL in 2003. Nucleic Acids Res. 2003;31(1):365–70.

38. Mistry J, Finn RD, Eddy SR, Bateman A, Punta M. Challenges in homology search: HMMER3 and convergent evolution of coiled-coil regions. Nucleic Acids Res. 2013;41(12):e121.

39. Zdobnov EM, Apweiler R. InterProScan–an integration platform for the signature-recognition methods in InterPro. Bioinformatics. 2001;17:847–8.

40. Conesa A, Götz S, García-Gómez JM, Terol J, Talón M, Robles M. Blast2GO: a universal tool for annotation, visualization and analysis in functional genomics research. Bioinformatics. 2005;21(18):3674–6.

41. She R, Chu JS, Wang K, Pei J, Chen N. GenBlastA: enabling BLAST to identify homologous gene sequences. Genome Res. 2009;19(1):143–9.

42. Birney E, Clamp M, Durbin R. GeneWise and genomewise. Genome Res. 2004;14(5):988–95.

43. Langmead B, Trapnell C, Pop M, Salzberg SL. Ultrafast and memory-efficient alignment of short DNA sequences to the human genome. Genome Biol. 2009;10(3):R25.

44. Bickhart DM, Rosen BD, Koren S, Sayre BL, Hastie AR, Chan S, et al. Single-molecule sequencing and chromatin conformation capture enable *de novo* reference assembly of the domestic goat genome. Nat Genet. 2017;49(4):643–50.

45. Garrison E, Marth G. Haplotype-based variant detection from short-read sequencing. ArXiv Prepr. 2012. ArXiv:1207.3907.

46. Kent WJ. BLAT–the BLAST-like alignment tool. Genome Res. 2002;12(4):656–64.

47. Simao FA, Waterhouse RM, Ioannidis P, Kriventseva EV, Zdobnov EM. BUSCO: assessing genome assembly and annotation completeness with single-copy orthologs. Bioinformatics. 2015;31(19):3210–2.

48 Gao F, Wang X, Li X, Xu M, Li H, Abla M et al. Supporting data for "Long-read sequencing and de novo genome assembly of Ammopiptanthus nanus, a desert shrub". GigaScience Database 2018. http://dx.doi.org/10.5524/100466

Tables

Table 1 Summary of *A. nanus* genome annotation

Method	Software and gene set	Gene number
Ab initio based	Genscan	26,702
	Augustus	43,844
	GlimmerHMM	42,368
	GeneID	45,561
	SNAP	55,094
Homology based	GeMoMa	
	Arachis duranensis	27,630
	Cicer arietinum	29,229
	Phaseolus vulgaris	27,554
	Glycine max	31,559
RNA-seq based	PASA	43,810
	TransDecoder	68,687
	GeneMarkS-T	44,944
Integration	EVM	37,173

Table 2 Summary of functional annotation for the predicted genes

Annotation database	Annotated gene number	Percentage (%)
GO	20,177	54.28
KEGG	10,130	27.25
KOG	18,237	49.06
Pfam	26,727	71.90
Swissprot	21,401	57.57
TrEMBL	34,946	94.01
NR	34,909	93.91
Nt	34,041	91.57
All Annotated	35,950	96.71

Table 3 The alignment of the unigenes to the *A. nanus* genome assembly

Range of length	Total number	Aligned number	Percentage (%)
≥500	81,429	81,429	100
≥1,000	54,385	54,385	100

Figure legends

Figure 1. A flowering *A. nanus*

Figure 1

Click here to access/download;Figure;Figure_1.jpg

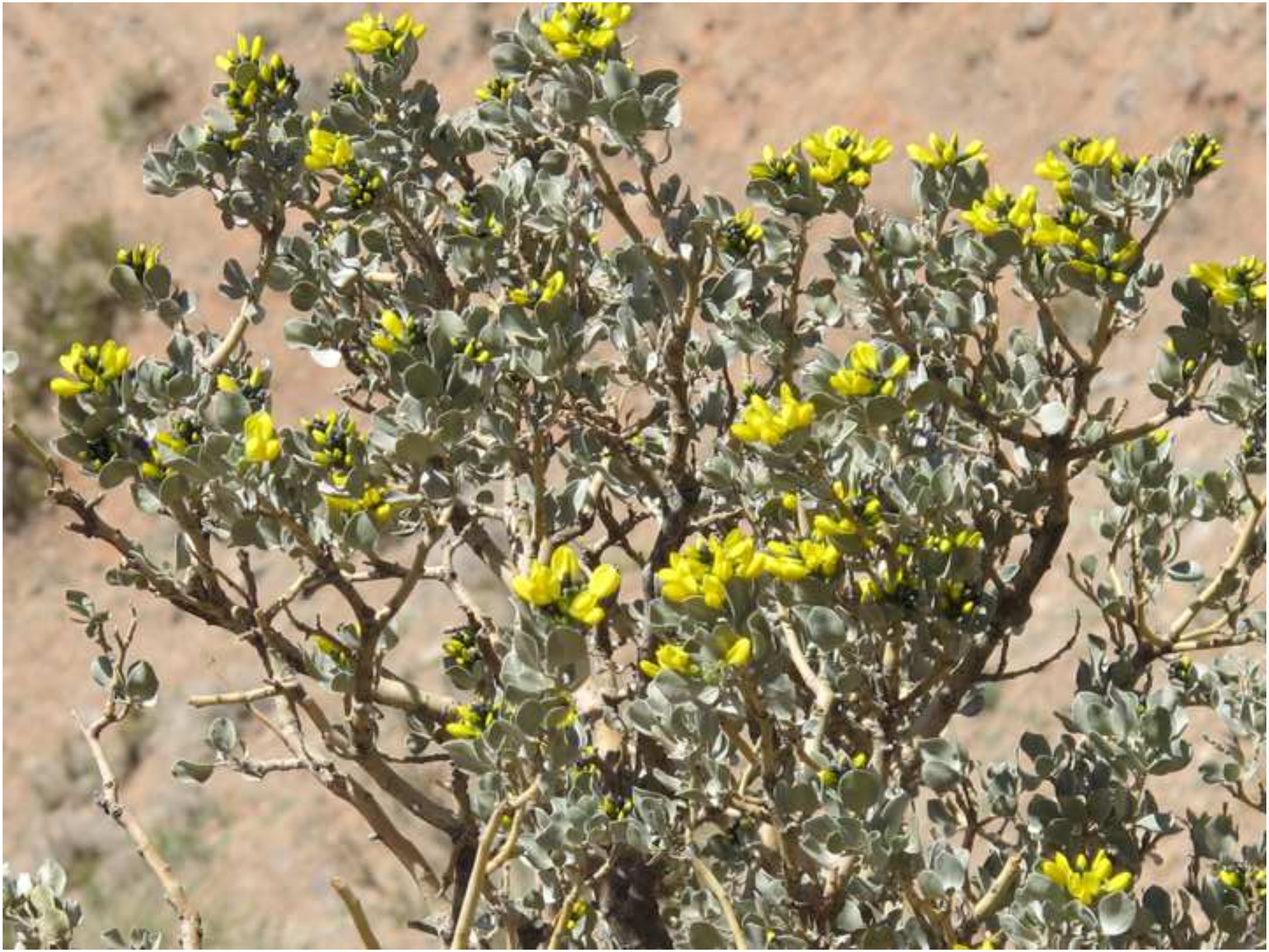

Click here to access/download
Supplementary Material
Supplementary material_20180322_10_8.docx
