# Supplementary material for: Long-read sequencing and de novo genome assembly of Ammopiptanthus nanus, a desert shrub
Source: Gigascience. 2018 Jun 28;7(7):giy074. doi: 10.1093/gigascience/giy074 (PMC6048559; doi:10.1093/gigascience/giy074)
Supplement: Additional Files [file giy074_supplement_files.docx]

**Supplementary material**

**For “Long-read sequencing and *de novo* genome assembly of *Ammopiptanthus nanus*, a desert shrub”**

**Table S1 Illumina sequencing reads used for genome size estimation and correction of genome assembly**

| Library | Data (Gb) | Depth (×) | Q20 (%) | Q30 (%) |
| --- | --- | --- | --- | --- |
| 350 bp | 55.97 | 62.96 | 96.65 | 91.67 |
| Total | 55.97 | 62.96 | - | - |

**Table S2 Size distribution of the PacBio subreads**

| Length (bp) | Number | Total length (bp) | Average length (bp) |
| --- | --- | --- | --- |
| 0~2000 | 1,446,242 | 1,726,833,856 | 11194.01 |
| 2000~4000 | 1,325,856 | 3,907,347,718 | 2947.04 |
| 4000~6000 | 1,025,450 | 5,090,549,348 | 4964.21 |
| 6000~8000 | 838,429 | 5,842,576,857 | 6968.48 |
| 8000~10000 | 697,322 | 6,255,043,413 | 8970.09 |
| 10000~12000 | 609,493 | 6,694,289,324 | 10983.37 |
| 12000~14000 | 532,230 | 6,900,418,300 | 12965.11 |
| 14000~16000 | 413,714 | 6,185,725,069 | 14951.69 |
| 16000~18000 | 306,204 | 5,188,857,740 | 16945.75 |
| 18000~ | 723,382 | 16,746,376,775 | 23150.12 |
| Total | 7,918,322 | 64,538,018,400 | 8150.47 |

**Table S3. Statistics of the *A. nanus* assembly**

| Contig number | Contig length (bp) | Contig N50 (bp) | Contig N90 (bp) | Contig max (bp) | GC content (%) | Gap total length (bp) |
| --- | --- | --- | --- | --- | --- | --- |
| 1,099 | 823,736,225 | 2,761,375 | 562,765 | 11,314,766 | 36.73 | 0 |

**Table S4 Statistics of the identified repeat sequences**

| Type | Number | Length (bp) | Percentage (%) |
| --- | --- | --- | --- |
| ClassI/DIRS | 83,795 | 69,770,021 | 8.47 |
| ClassI/LINE | 81,457 | 24,796,391 | 3.01 |
| ClassI/LTR | 66,185 | 46,144,753 | 5.6 |
| ClassI/LTR/Copia | 207,910 | 147,578,032 | 17.91 |
| ClassI/LTR/Gypsy | 235,097 | 246,456,299 | 29.92 |
| ClassI/PLE\|LARD | 235,732 | 88,032,322 | 10.69 |
| ClassI/SINE | 4,694 | 793,630 | 0.1 |
| ClassI/SINE\|TRIM | 2 | 2,509 | 0 |
| ClassI/TRIM | 2,346 | 3,722,901 | 0.45 |
| ClassI/Unknown | 336 | 72,148 | 0.01 |
| ClassII/Crypton | 8 | 377 | 0 |
| ClassII/Helitron | 10,187 | 3,723,094 | 0.45 |
| ClassII/MITE | 26,586 | 4,955,711 | 0.6 |
| ClassII/Maverick | 3,758 | 1,462,846 | 0.18 |
| ClassII/TIR | 139,094 | 66,281,083 | 8.05 |
| ClassII/Unknown | 4,689 | 1,095,615 | 0.13 |
| PotentialHostGene | 18,664 | 4,149,732 | 0.5 |
| SSR | 4,441 | 924,845 | 0.11 |
| Unknown | 122,377 | 44,200,761 | 5.37 |
| Unknown/Helitron\|LARD/? | 39 | 16,961 | 0 |
| Total without overlap | 1,247,397 | 610,249,749 | 74.08 |

**Table S5 Statistics of mapping the transcriptome data to genome assembly**

| Exon-mapped ratio | Intron-mapped ratio | Intergenic region-mapped ratio |
| --- | --- | --- |
| 89.05% | 5.26% | 5.70% |

**Table S6 Statistics of the genome annotation**

| Gene number | Gene Length (bp) | Average gene length (bp) | CDS length (bp) | Average CDS length (bp) | Intron length (bp) | Average intron length (bp) |
| --- | --- | --- | --- | --- | --- | --- |
| 37,188 | 166,889,308 | 4,487.72 | 41,955,439 | 1,128.20 | 124,933,869 | 3359.52 |

**Table S7 Statistics of the predicted pseudogenes**

| Pseudogene number | Total length (bp) | Average length (bp) |
| --- | --- | --- |
| 7,891 | 20,960,968 | 2656.31 |

**Table S8 The alignment of the Illunima reads to the *A. nanus* genome assembly**

| Library | Total reads | Mapped (%) | Concordantly mapped (%) |
| --- | --- | --- | --- |
| 350 bp | 365,508,866 | 100 | 98.45 |

**Table S9 BUSCO assessment of the *A. nanus* genome assembly**

| Complete BUSCOs | Complete and single-Copy BUSCOs | Complete and Duplicated BUSCOs | Fragmented BUSCOs | Missing BUSCOs |
| --- | --- | --- | --- | --- |
| 1,327 | 1,238 | 89 | 37 | 76 |


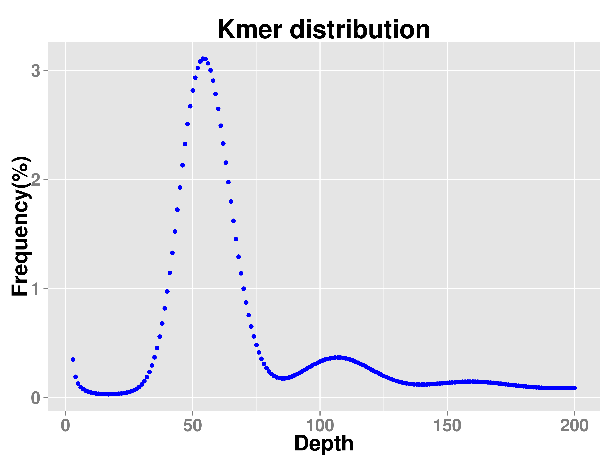


**Figure S1. Distribution of k-mers of length 19 from the Illumina Hiseq reads**


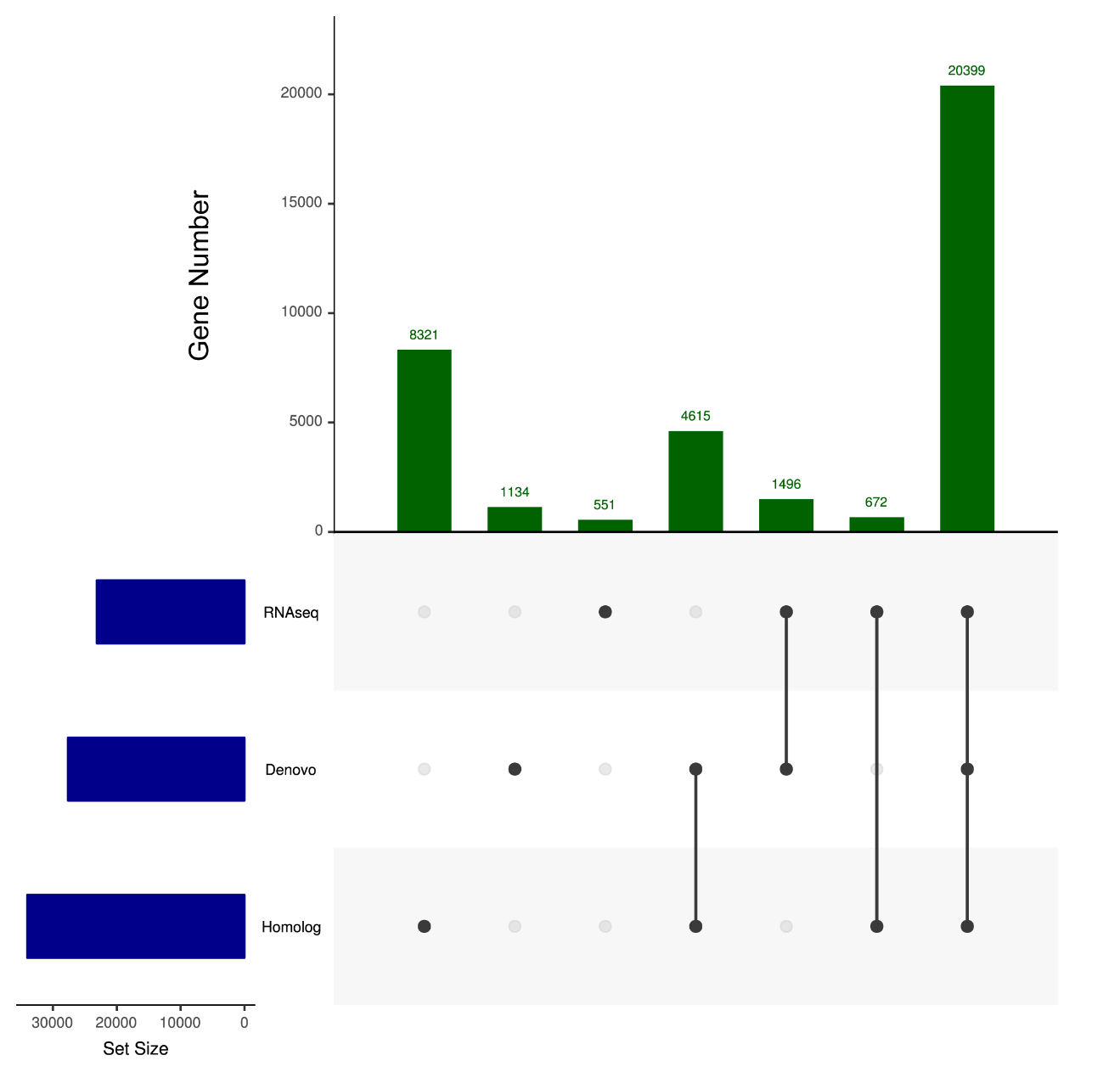


**Figure S2**. Venn diagram plot using UpSetR showing the overlap of the gene annotation results of the three gene prediction methods
