# Supplementary material for: Long-read sequencing and de novo genome assembly of Ammopiptanthus nanus, a desert shrub
Source: Gigascience. 2018 Jun 28;7(7):giy074. doi: 10.1093/gigascience/giy074 (PMC6048559; doi:10.1093/gigascience/giy074)
Supplement: Reviewer_1_Report_(Original_Submission) -- Nevin Young — 11/13/2017 Reviewed [file giy074_reviewer_1_report_(original_submission).pdf]

Reviewer Report

Title: Long-read sequencing and de novo genome assembly of Ammopiptanthus nanus, a desert shrub

Version: Original Submission **Date:** 11/13/2017

Reviewer name: Nevin Young

Reviewer Comments to Author:

This report describes a standard, PacBio-based sequence and assembly for Ammonpiptanthus. The underlying sequencing technology is reasonable, apparently involving DNA template of acceptable length and quality and leading to sequence reads and depth suitable for whole genome assembly. Subsequent analyses (k-mer, repeat, genome feature annotation) are standard with methods described in sufficient detail. Accompanying files are typical and sufficient for most follow-up analyses researchers may pursue. However, there are numerous English grammar errors in the text that should be corrected. Citation(s) supporting the statement that the genus is interesting and characterized in terms of abiotic stress tolerance are missing (that is, plant physiology research, not just transcriptomic analyses). Non-genomic research supporting the very low level of heterozygosity, should accompany the value reported here. Finally, locations in the text where accompanying data files are available should be highlighted within the text.

Level of Interest

Please indicate how interesting you found the manuscript: An article whose findings are important to those with closely related research interests

Quality of Written English

Please indicate the quality of language in the manuscript: Needs some language corrections before being published

Declaration of Competing Interests

Please complete a declaration of competing interests, considering the following questions:

- Have you in the past five years received reimbursements, fees, funding, or salary from an organisation that may in any way gain or lose financially from the publication of this manuscript, either now or in the future?
- Do you hold any stocks or shares in an organisation that may in any way gain or lose financially from the publication of this manuscript, either now or in the future?
- Do you hold or are you currently applying for any patents relating to the content of the manuscript?
- Have you received reimbursements, fees, funding, or salary from an organization that holds or has applied for patents relating to the content of the manuscript?
- Do you have any other financial competing interests?

- Do you have any non-financial competing interests in relation to this paper?

If you can answer no to all of the above, write 'I declare that I have no competing interests' below. If your reply is yes to any, please give details below.

I declare that I have no competing interests.

I agree to the open peer review policy of the journal. I understand that my name will be included on my report to the authors and, if the manuscript is accepted for publication, my named report including any attachments I upload will be posted on the website along with the authors' responses. I agree for my report to be made available under an Open Access Creative Commons CC-BY license (http://creativecommons.org/licenses/by/4.0/). I understand that any comments which I do not wish to be included in my named report can be included as confidential comments to the editors, which will not be published.

I agree to the open peer review policy of the journal

To further support our reviewers, we have joined with Publons, where you can gain additional credit to further highlight your hard work (see: https://publons.com/journal/530/gigascience). On publication of this paper, your review will be automatically added to Publons, you can then choose whether or not to claim your Publons credit. I understand this statement.Yes
