# Supplementary material for: Long-read sequencing and de novo genome assembly of Ammopiptanthus nanus, a desert shrub
Source: Gigascience. 2018 Jun 28;7(7):giy074. doi: 10.1093/gigascience/giy074 (PMC6048559; doi:10.1093/gigascience/giy074)
Supplement: Reviewer_2_Report_(Original_Submission) -- Yinping Jiao — 11/19/2017 Reviewed [file giy074_reviewer_2_report_(original_submission).pdf]

Reviewer Report

Title: **Long-read sequencing and de novo genome assembly of Ammopiptanthus nanus, a desert shrub**

Version: **Original Submission** **Date:** 11/19/2017

Reviewer name: **Yinping Jiao**

Reviewer Comments to Author:

This manuscript presented the genome assembly and annotation of Ammopiptanthus nanus. This dataset could add more resources for plant genomics, especially for the dry land plant research. Below are my detailed concerns:1) As a genomics resource, the authors didn't release the data properly. Even though the raw sequencing reads are in SRA, a lot of future data users would look for the genome assembly and annotation. A genome browser and ftp will be requested. Reference genome would request further improvement and maintenance. I would suggest the authors to have a genome browser up or deposit the genome to the large databases like NCBI, Ensembl-plants. In this way, users would have better access to the genome. On the hand, the feedback from the users could help to improve this geneome.2) As a manuscript it is not well organized. There are seven tables, five of which only have one row except for the header. These numbers have been described in the text. These tables should be reorganized.3) It doesn't make a lot of sense to have a main figure to only show the look of the plant. As the author tried to introduce this species, an evolutionary species tree would be more informative.4) The last paragraph about the background (Line 61-63): "Most of the de novo assemblies of plant genomes reported recently have been performed using the next generation sequencing technologies such as Illumina or 454 sequencing platforms" is too subjective, as the authors would like to highlight their genome was done by PacBio. Recently there is also a list of complicated plant genomes being done by third generation sequencing: maize, sunflower, Oropetium thomaeum, rice, Chenopodium quinoa, thomaeum. Authors should not ignore these high genomes. They should be included in introduction.5) After the genome assembly, the contigs were only polished by Pilon using short reads. I would also recommend to also use PacBio reads to correct the base calling, as illumina reads may not be able to cover all the contigs due to alignment and sequencing bias. The number of bases were checked and corrected should be mentioned, too.6) Authors deployed several approaches for gene annotation. Figure 2 is not very informative. A flowchart figure describing how the gene annotation was done could be better. I also have concerns that only default parameters from the gene predictors were used for ab initio prediction. Many of these default prediction parameters were not suitable for plants. They request training models to generate accurate gene models. A comparative analysis with close related species will be strong evidence to show the quality of gene models. With the current data, there is no evaluation of the quality of gene models.

Level of Interest

Please indicate how interesting you found the manuscript: An article whose findings are important to those with closely related research interests

Quality of Written English

Please indicate the quality of language in the manuscript: Not suitable for publication unless extensively edited

Declaration of Competing Interests

Please complete a declaration of competing interests, considering the following questions:

- Have you in the past five years received reimbursements, fees, funding, or salary from an organisation that may in any way gain or lose financially from the publication of this manuscript, either now or in the future?
- Do you hold any stocks or shares in an organisation that may in any way gain or lose financially from the publication of this manuscript, either now or in the future?
- Do you hold or are you currently applying for any patents relating to the content of the manuscript?
- Have you received reimbursements, fees, funding, or salary from an organization that holds or has applied for patents relating to the content of the manuscript?
- Do you have any other financial competing interests?
- Do you have any non-financial competing interests in relation to this paper?

If you can answer no to all of the above, write 'I declare that I have no competing interests' below. If your reply is yes to any, please give details below.

I declare that I have no competing interests

I agree to the open peer review policy of the journal. I understand that my name will be included on my report to the authors and, if the manuscript is accepted for publication, my named report including any attachments I upload will be posted on the website along with the authors' responses. I agree for my report to be made available under an Open Access Creative Commons CC-BY license (http://creativecommons.org/licenses/by/4.0/). I understand that any comments which I do not wish to be included in my named report can be included as confidential comments to the editors, which will not be published.

I agree to the open peer review policy of the journal
