# Supplementary material for: Long-read sequencing and de novo genome assembly of Ammopiptanthus nanus, a desert shrub
Source: Gigascience. 2018 Jun 28;7(7):giy074. doi: 10.1093/gigascience/giy074 (PMC6048559; doi:10.1093/gigascience/giy074)
Supplement: Reviewer_2_Report_(Revision_1) -- Yinping Jiao — 01/18/2018 Reviewed [file giy074_reviewer_2_report_(revision_1).pdf]

Reviewer Report

Title: **Long-read sequencing and de novo genome assembly of Ammopiptanthus nanus, a desert shrub**

Version: **Revision 1** **Date:** 1/18/2018

Reviewer name: **Yinping Jiao**

Reviewer Comments to Author:

The authors have clarified important issues and questions raised by the reviewers and added helpful citations. Minor points: 1. Even though the authors stated that they have deposited the data as Gigascience requested, an additional genome browser will be still very helpful for users to navigate this genome as a reference. I would suggest the authors to work with other plant genomics databases (PlantDB, Gramene and so on) to make their genome more accessible to users.2. Line 27-28: BUSCO can only estimate the completeness of gene space. The statement of "the genome completeness" is inaccurate. Please revise.3. Line 65: N50 is only a statistic to measure assemblies. Please revise this sentence to "these assemblies generally content very fragmented sequences", or something similar.

Level of Interest

Please indicate how interesting you found the manuscript: An article whose findings are important to those with closely related research interests

Quality of Written English

Please indicate the quality of language in the manuscript: Acceptable

Declaration of Competing Interests

Please complete a declaration of competing interests, considering the following questions:

- Have you in the past five years received reimbursements, fees, funding, or salary from an organisation that may in any way gain or lose financially from the publication of this manuscript, either now or in the future?
- Do you hold any stocks or shares in an organisation that may in any way gain or lose financially from the publication of this manuscript, either now or in the future?
- Do you hold or are you currently applying for any patents relating to the content of the manuscript?
- Have you received reimbursements, fees, funding, or salary from an organization that holds or has applied for patents relating to the content of the manuscript?
- Do you have any other financial competing interests?
- Do you have any non-financial competing interests in relation to this paper?

If you can answer no to all of the above, write 'I declare that I have no competing interests' below. If your reply is yes to any, please give details below.

I declare that I have no competing interests

I agree to the open peer review policy of the journal. I understand that my name will be included on my report to the authors and, if the manuscript is accepted for publication, my named report including any attachments I upload will be posted on the website along with the authors' responses. I agree for my report to be made available under an Open Access Creative Commons CC-BY license (http://creativecommons.org/licenses/by/4.0/). I understand that any comments which I do not wish to be included in my named report can be included as confidential comments to the editors, which will not be published.

I agree to the open peer review policy of the journal

To further support our reviewers, we have joined with Publons, where you can gain additional credit to further highlight your hard work (see: https://publons.com/journal/530/gigascience). On publication of this paper, your review will be automatically added to Publons, you can then choose whether or not to claim your Publons credit. I understand this statement. Yes
