# Supplementary material for: Long-read sequencing and de novo genome assembly of Ammopiptanthus nanus, a desert shrub
Source: Gigascience. 2018 Jun 28;7(7):giy074. doi: 10.1093/gigascience/giy074 (PMC6048559; doi:10.1093/gigascience/giy074)
Supplement: Reviewer_3_Report_(Original_Submission) -- Felix Bemm — 11/27/2017 Reviewed [file giy074_reviewer_3_report_(original_submission).pdf]

Reviewer Report

Title: Long-read sequencing and de novo genome assembly of Ammopiptanthus nanus, a desert shrub

Version: Original Submission **Date:** 11/27/2017

Reviewer name: Felix Bemm

Reviewer Comments to Author:

The article (Manuscript#: GIGA-D-17-00264) entitled "Long-read sequencing and de novo genome assembly of Ammopiptanthus nanus, a desert shrub", by Fei Gao and collaborators delivers "a de novo assembly of the rare broad-leaved shrub Ammopiptanthus nanus". The work presents the completeness of the assembled genome as well its gene and repeat annotation. It's major finding is the high amonunt of repetitive elements and the authors suggest their work as a valuable source for comparative genomics analysis in the family of legumes. I agree with most of what the authors state but have the following suggestions:1) The size of the genome was estimated via k-mer distribution. Figure S1 shows a major and a minor peak. I agree with the authors that the highest peak represents the diploid genome given the low amounts of BUSCO duplicates. Nevertheless, a in-depth description of the repetitve peak (better contigs associated with it) would be interesting and should be added to the manuscript. Please describe how much contigs and how much total sequence are duplicated (showing k-mers from the second peak) and elaborate on the annotated genes/repeats you can find of there. Consider moving the k-mer histogram and maybe a graphical (GO enrichment based) summary of duplicated genes/repeats to the main text as figure.2) The genome was assembled with Canu and polished with Pilon. Could the authors explain while the initial Canu assembly was not polished with Arrow prior to Pilon polishing. A lot of medium sized InDels usually remain in the assembly if only Pilon is used for assembly polishing. The final genome release should include a Arrow polishing step. The genome assembly should be deposited in one of the public databases.3) Repeats and genes were predicted with various tools. The text is missing details on which models the ab initios gene predictors were used with (e.g., Augustus, SNAP). Please describe how these models were generated.4) Line 145-148: Please remove the term "functionally annotated" and repleace it with something appropriate (e.g., classified into families and predicting domains and important sites). The tools mentioned are annotating evolutionary conserved domains or lift over putative functions via homology-based methods but none of them are able to annotate genes functionally.5) Please add a short section/sentence about the gene models that were choosen by EVM. Simply indicate which was the prefered gene prediction tool/model that EVM selected from.6) Please indicate whether default mapping parameteres were used to assess the genome completeness via short read mappings and adopt the quality assessment method from (Bickhart et al. 2017, Nat Genet.; Jain et al. 2017, bioRxiv).7) Please indicate that the data used for Pilon polishing is the same that is used during the completeness assessment and state any eventual bias.6) Please replace Figure 2 with either a scaled Venn diagram but better with a UpSetR plot to ease interpretation of the gene prediction integration. Further consider moving the Figure 2 to the supplement since Table 3 is more than enough.

Level of Interest

Please indicate how interesting you found the manuscript: An article whose findings are important to those with closely related research interests

Quality of Written English

Please indicate the quality of language in the manuscript: Not suitable for publication unless extensively edited

Declaration of Competing Interests

Please complete a declaration of competing interests, considering the following questions:

- Have you in the past five years received reimbursements, fees, funding, or salary from an organisation that may in any way gain or lose financially from the publication of this manuscript, either now or in the future?
- Do you hold any stocks or shares in an organisation that may in any way gain or lose financially from the publication of this manuscript, either now or in the future?
- Do you hold or are you currently applying for any patents relating to the content of the manuscript?
- Have you received reimbursements, fees, funding, or salary from an organization that holds or has applied for patents relating to the content of the manuscript?
- Do you have any other financial competing interests?
- Do you have any non-financial competing interests in relation to this paper?

If you can answer no to all of the above, write 'I declare that I have no competing interests' below. If your reply is yes to any, please give details below.

I declare that I have no competing interests.

I agree to the open peer review policy of the journal. I understand that my name will be included on my report to the authors and, if the manuscript is accepted for publication, my named report including any attachments I upload will be posted on the website along with the authors' responses. I agree for my report to be made available under an Open Access Creative Commons CC-BY license (http://creativecommons.org/licenses/by/4.0/). I understand that any comments which I do not wish to be included in my named report can be included as confidential comments to the editors, which will not be published.

I agree to the open peer review policy of the journal

To further support our reviewers, we have joined with Publons, where you can gain additional credit to further highlight your hard work (see: https://publons.com/journal/530/gigascience). On publication of this paper, your review will be automatically added to Publons, you can then choose whether or not to claim your Publons credit. I understand this statement. Yes
