# Supplementary material for: Long-read sequencing and de novo genome assembly of Ammopiptanthus nanus, a desert shrub
Source: Gigascience. 2018 Jun 28;7(7):giy074. doi: 10.1093/gigascience/giy074 (PMC6048559; doi:10.1093/gigascience/giy074)
Supplement: Reviewer_3_Report_(Revision_1) -- Felix Bemm — 8/02/2018 Reviewed [file giy074_reviewer_3_report_(revision_1).pdf]

Reviewer Report

Title: **Long-read sequencing and de novo genome assembly of Ammopiptanthus nanus, a desert shrub**

Version: **Revision 1** **Date:** 2/8/2018

Reviewer name: **Felix Bemm**

Reviewer Comments to Author:

The revised manuscript addressed most of my comments properly. Two major and one minor concerns however remain open.2) The genome was assembled with Canu and polished with Pilon. Could the authors explain while the initial Canu assembly was not polished with Arrow prior to Pilon polishing. A lot of medium sized InDels usually remain in the assembly if only Pilon is used for assembly polishing. The final genome release should include a Arrow polishing step.The authors response to my comment and a similar one from reviewer 2 (5th comment) is not satisfying. Yes, long read polishing is not increasing assembly quality to the same extent as a final short read polishing. But long read polishing removes spurious insertions and deletions at a length were short reads fail. The authors obviously did not understand that each polishing method addresses different types of assembly errors and therefore both methods should be applied in modern genomes projects, beginning with long reads and ending with short reads. The manuscript should specifically state that a long read polishing was not applied, and longer spurious insertion and deletions introduced during assembly might not be corrected. 6) Please indicate whether default mapping parameters were used to assess the genome completeness via short read mappings and adopt the quality assessment method from (Bickhart et al. 2017, Nat Genet.; Jain et al. 2017, bioRxiv).The second concern is the error evaluation analysis the authors applied. They did not what I asked for and/or misunderstood the Bickhart et al. 2017, Nat Genet. reference I added. Authors simply reported re-mapping ratios instead of calling SNPs with the re-mappings and inferring Q-value as pointed out by Bickhart et al. The manuscript should contain a sentence that only completeness was tested but accuracy of the assembly was not specifically assessed or, and that would be my preferred option, the authors should re-run the analysis. A simple workflow can be taken from https://github.com/fbemm/onefc-oneasm/wiki/Assembly-Validation.3) The size of the genome was estimated via k-mer distribution. Figure S1 shows a major and a minor peak. I agree with the authors that the highest peak represents the diploid genome given the low amounts of BUSCO duplicates. Nevertheless, a in-depth description of the repetitve peak (better contigs associated with it) would be interesting and should be added to the manuscript. Please describe how much contigs and how much total sequence are duplicated (showing k-mers from the second peak) and elaborate on the annotated genes/repeats you can find of there. Consider moving the k-mer histogram and maybe a graphical (GO enrichment based) summary of duplicated genes/repeats to the main text as figure.Authors misunderstood my request. The idea was, to isolates k-Mers from the k-Mer distribution (can be simply done with Jellyfish dump by specifying a low and high cutoff that fits to the 2nd peak) and use them as baits to isolate contigs that contain these k-Mer (can be done using "bbduk2 in=contigs.fasta out=baited.fasta ref=2nd-peak-kmers-fasta hdist=0 mm=f). It would have been interesting to see if these elements are a) properly assembled, thus appear on contigs with regions from the first peak or b) are simply collapsed during the assembly. On top of that an repeat/annotation analysis could have revealed the nature of the contigs and thereby have helped to understand any functional implication of this partial genome duplication. It is up to the authors to add that analysis, but it would have improved the understand of the A. nanus genome for sure.

Level of Interest

Please indicate how interesting you found the manuscript: An article of importance in its field

Quality of Written English

Please indicate the quality of language in the manuscript: Acceptable

Declaration of Competing Interests

Please complete a declaration of competing interests, considering the following questions:

- Have you in the past five years received reimbursements, fees, funding, or salary from an organisation that may in any way gain or lose financially from the publication of this manuscript, either now or in the future?
- Do you hold any stocks or shares in an organisation that may in any way gain or lose financially from the publication of this manuscript, either now or in the future?
- Do you hold or are you currently applying for any patents relating to the content of the manuscript?
- Have you received reimbursements, fees, funding, or salary from an organization that holds or has applied for patents relating to the content of the manuscript?
- Do you have any other financial competing interests?
- Do you have any non-financial competing interests in relation to this paper?

If you can answer no to all of the above, write 'I declare that I have no competing interests' below. If your reply is yes to any, please give details below.

I declare that I have no competing interests.

I agree to the open peer review policy of the journal. I understand that my name will be included on my report to the authors and, if the manuscript is accepted for publication, my named report including any attachments I upload will be posted on the website along with the authors' responses. I agree for my report to be made available under an Open Access Creative Commons CC-BY license (http://creativecommons.org/licenses/by/4.0/). I understand that any comments which I do not wish to be included in my named report can be included as confidential comments to the editors, which will not be published.

I agree to the open peer review policy of the journal

To further support our reviewers, we have joined with Publons, where you can gain additional credit to further highlight your hard work (see: https://publons.com/journal/530/gigascience). On publication of this paper, your review will be automatically added to Publons, you can then choose whether or not to claim your Publons credit. I understand this statement. Yes
